# Review: performance of jujube and its extracts in cancer: therapeutic, toxicity-reducing and potentiating effects

**DOI:** 10.3389/fonc.2025.1489974

**Published:** 2025-01-29

**Authors:** Boyun Gou, Guoqing Chen, Shicong Huang, Na Ning, Qian Gu, Shuai Duan, Yuhua Du, Yi Nan, Ling Yuan

**Affiliations:** ^1^ College of Pharmacy, Ningxia Medical University, Yinchuan, Ningxia Hui Autonomous Region, China; ^2^ Key Laboratory of Ningxia Ethnomedicine Modernization, Ministry of Education, Ningxia Medical University, Yinchuan, Ningxia Hui Autonomous Region, China

**Keywords:** jujube, homology of medicine and food, anti-cancer, adverse reactions, extraction and separation, attenuated toxicity and potentiating effects

## Abstract

Cancer is one of the most serious public health challenges in the world. The number of new and fatal patients with cancer continues to increase every year, which poses a serious threat to human health. Although there are effective treatments such as radiotherapy and chemotherapy for cancers, they are often accompanied by serious side effects. With the development of the pharmaceutical industry, there is a gradual desire to develop low-toxicity and effective anticancer drugs from traditional Chinese herbs. Due to its “homology of medicine and food,” jujube is one of the traditional Chinese herbs that is extensively used in China. In addition, jujube has received much attention around the world for its excellent pharmacological effects and food value. This article reviews the anticancer effects of a fruit, food, and drug, jujube, which have been shown in current studies, and analyzes its therapeutic mechanism, active ingredients, extraction and isolation methods, as well as its synergistic and attenuation performance in cancer. This will further promote the progress of natural medicine’s anti-cancer effect.

## 1 Introduction

Cancer is one of the most serious public health challenges facing the world today, with 27 million new cases predicted by 2040 (1). In addition, cancer kills up to 25% of patients every year (2), which is still a great threat to human health. The current treatment of cancer can be roughly divided into four categories: (1) surgical removal of cancer cells; (2) killing cancer cells by some chemotherapeutic drugs and cancer-specific drugs; (3) radiotherapy; (4) spontaneous regression of cancer cells (3).

These treatments, while effective against cancers, also result in significant collateral damage to normal cells (4). Chemotherapy drugs are often associated with severe side effects and high costs. Scientists are paying more and more attention to the study of natural products. Natural products used to treat and prevent cancer have become a new way for drugs to fight cancer due to their unique molecular characteristics, excellent efficacy and safety (5).

In recent years, with increasing attention to food safety and health, there has been a growing focus on the health benefits and therapeutic effects of food (6). In traditional Chinese medicine, this is called “homology of medicine and food”. The concept of “homology of medicine and food” can be traced back to “Huangdi Neijing” (Huangdi’s Canon of Medicine), in which it was mentioned that “take empty food as food and take medicine to the patient”, which was the first time to put forward the concept that food can be medicine. In 500 A.D, the concept of diet therapy was first mentioned by the famous doctor Sun Simiao in his “Beiji Qianjin Yaofang” (Valuable Prescription for Emergency). Nowadays, with the continuous development of medicine and the accumulation of experience, people have discovered that drug-food homologous substances have significant advantages in reducing blood glucose, blood lipids, anti-oxidation (7), anti-inflammation (8), immune regulation (9) and anti-cancer (10). By 2022, 110 kinds of medicinal herbs with homology of medicine and food were listed by relevant state departments according to the Food Safety Law of the People’s Republic of China and a series of measures such as safety assessments (11).

**Figure 1:**
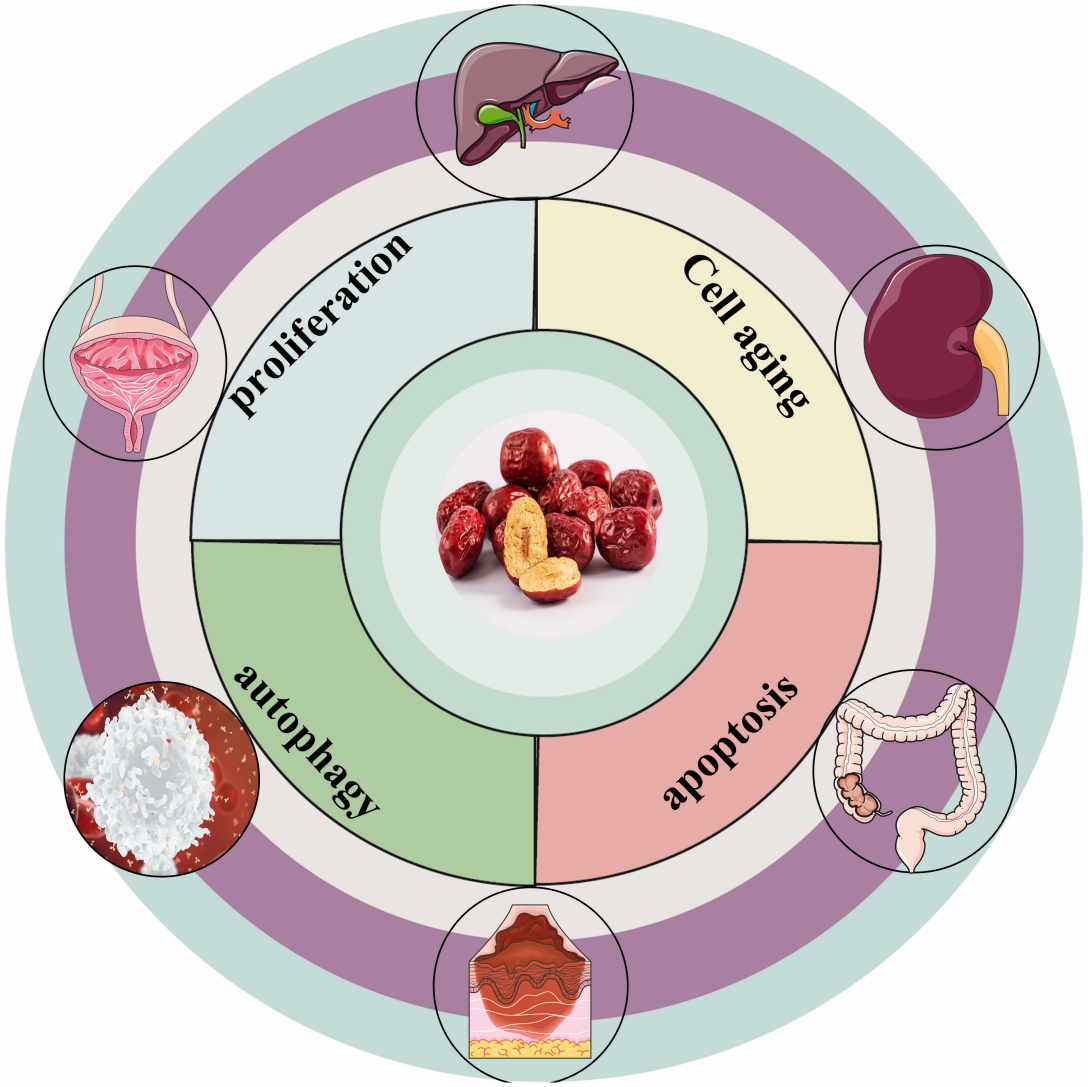
Types of cancers treated with jujube. Jujube has excellent therapeutic effects on liver, colorectum, cervical, renal, blood, melanoma cancers.

**Figure 2:**
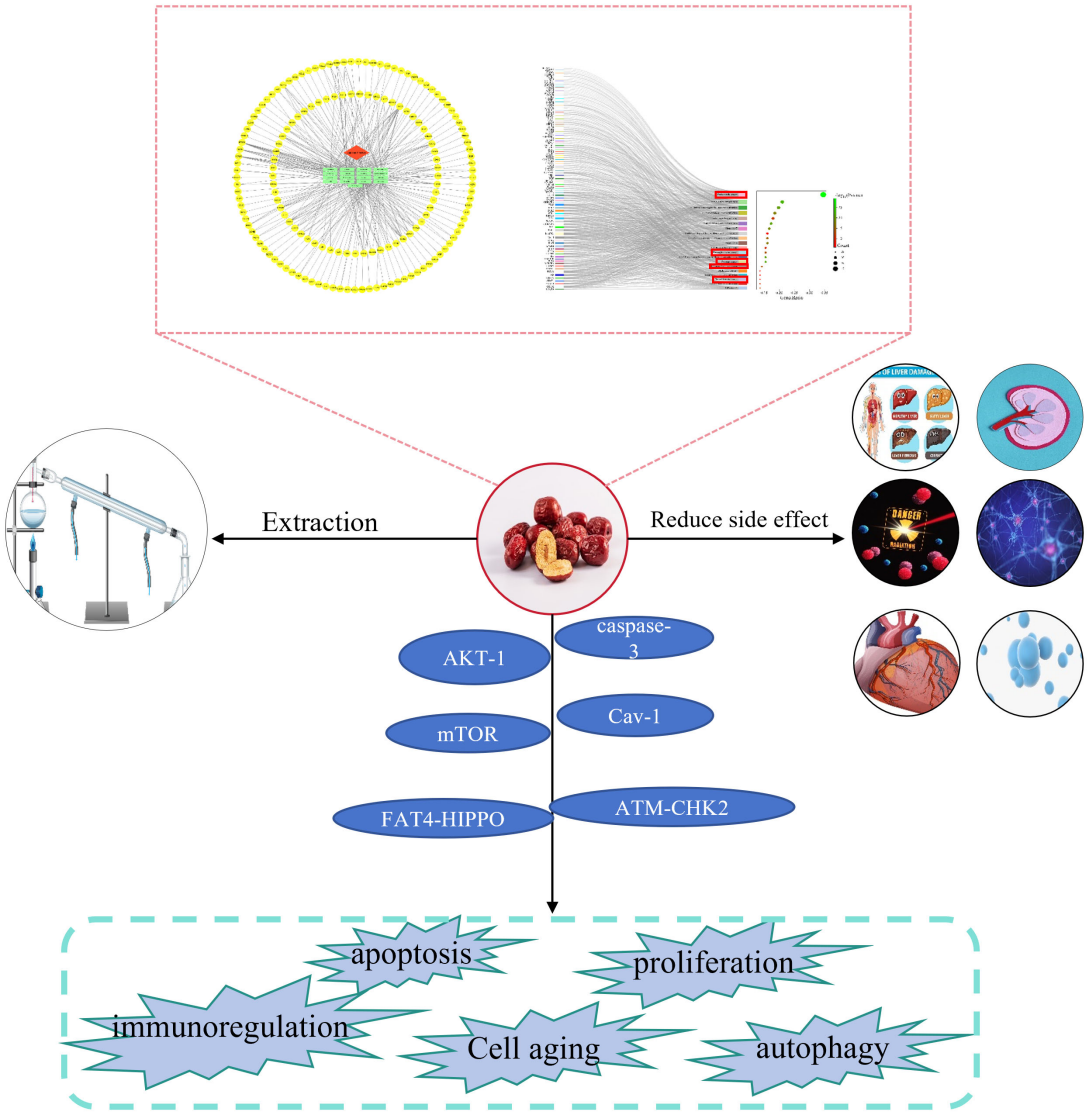
Flow diagram. The anticancer effect of jujube was analyzed by network pharmacology. The extraction and separation method, attenuation function, anti-cancer effect and mechanism of jujube were summarized. Pathways associated with cancer in the Kyoto Encyclopedia of Genes and Genomes are shown in the red box. AKT-1, caspase-3, mTOR, Cav-1, FAT4-HIPPO, and ATM-CHK2 are the signaling axes.

“Da Zao”(‘Ziziphus jujuba’ Mill.) is a nutritious and potent fruit (12). Jujube, the mature fruit of the Rhamnaceae family plant jujube is widely cultivated in Europe, Australia, South Asia, East Asia, and northern China (13). In particular, jujube has been used in China for thousands of years as a fruit, medicine, and food (14). In traditional Chinese medicine, jujube is commonly used as a treatment for anemia and hemorrhage (15). Globally, jujube has been discovered to have various pharmacological effects, including the treatment of anemia (15),anti-oxidation (16), immune regulation (17), anti-inflammation (18), anti-cancer (19), etc. Scientists have conducted in-depth research on jujube and discovered that jujube and its active ingredients have anti-cancer properties, which can effectively fight various types of cancers. **Figure 1** depicts the cancer types treated with jujube. This review summarizes jujube in the past 10 years and its active ingredient in anti-cancer and attenuated cancer treatment efficiency performance. It also provides an overview of the extraction and separation methods for its effective components. **Figure 2** is a flow chart.

## 2 Network pharmacology

**Figure 3:**
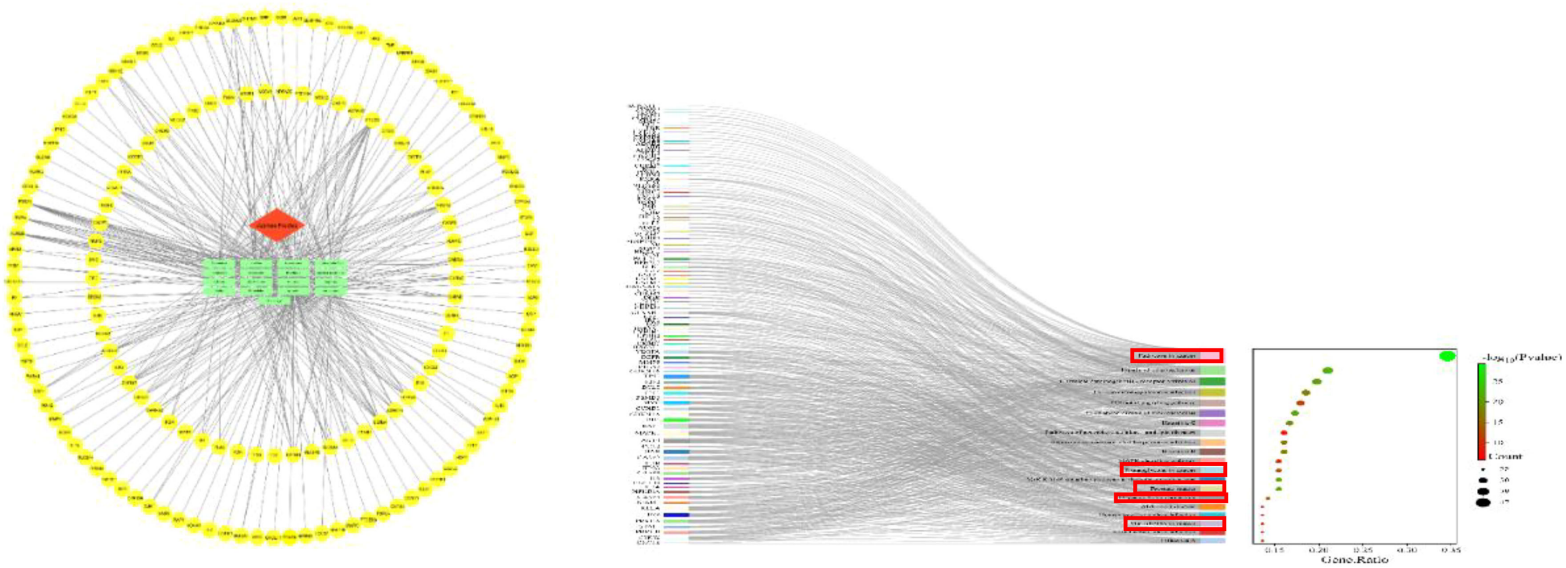
Jujube-active composition target plot and Kyoto Encyclopedia of Genes and Genomes analyses. In the red box are the signaling pathways associated with cancers in the Kyoto Encyclopedia of Genes and Genomes enrichment analysis.

To investigate the potential anticancer effects of jujube, a network pharmacology analysis was performed. This analysis takes into account the multi-component, multi-target, and multi-level nature of jujube. According to the criteria of oral bioavailability ≥30% and drug similarity ≥0.18, a total of 13 active ingredients were selected from the TCMSP database (https://old.tcmsp-e.com/tcmsp.php) (20). Protein names were entered into the multiprotein section of the STRING database (https://cn.string-db.org), specifically selecting the Homo sapientia species. The gene names corresponding to the protein names were then downloaded and matched (**Supplementary Table 2**). We used Cytoscape 3.9.1 to construct physiological network maps of the 13 active components and corresponding genes. In the figure, the red module represents the name of the medicinal herb, the blue template represents the active ingredient, and the yellow module represents the corresponding gene. We used DAVID database (https://david.ncifcrf.gov) on genes that were analyzed, and the use of bioinformatics online platform (http://www.bioinformatics.com.cn/) in the analysis result carried out the visualization processing (21). Results as shown in **Figure 3**, most of the genes are enriched in the cancer. This provides a theoretical basis for us to study the treatment of cancer with jujube.

## 3 Method of extraction

At present, the extraction of active ingredients from natural plants in a safe, green, and efficient manner is highly popular. As a trendy fruit and plant with notable anti-cancer activity, there have been many studies on the extraction of the active ingredients contained in jujube. Zhao (22)compared three different extraction methods when extracting phenolic substances in jujube and silymarin in jujube seeds, namely, conventional Soxhlete extraction, ultrasound-assisted extraction and supercritical fluid extraction, and finally certified that the efficiency of supercritical fluid extraction could be six times that of Soxhlete extraction. Zou et al. used an ultra-high-pressure assisted DES(Deep Eutectic Solvent) to extract JU-H,JU-U,JU-D and JU-UD, four polysaccharides of jujube (23). As one of the solvents that is recognized for its low pollution, high efficiency, and ability to be reused, DES is composed of hydrogen bond donor (HBD) and hydrogen bond acceptor (HBA). Under certain conditions, the acceptor and the donor can form a hydrogen bond to form a low utility solvent to extract the active components of jujube, and then only the hydrogen bond needs to be broken. The extracted active ingredients can be separated and the separation is completed. This solvent has been applied to most natural drugs and food, and has certain safety and reliability. Multiphase extraction also has certain advantages in extracting natural components, and its mild extraction environment can make the natural component activity almost unaffected. We summarized the popular methods for the extraction and separation of active ingredients in Chinese jujube. As shown in **Table 1**.

**Table 1:** The extraction method of jujube’s active ingredients.

Ingredients	Method	Solvent	Efficiency	References
Jujube polysaccharide	DES	Choline chloride (HBA), ethylene glycol (HBD)	10.42%	(23)
Flavonoids	Water, alcohol, microblog assisted extraction, DES	Water, methanol, choline chloride, citric acid, etc	8.03mg/g	(24)
Flavonoids	Aqueous two-phase extraction	Ethanol, K_2_HPO	6.53mg/g	(25)
Liquid jujube	Enzyme extraction	Cellulase	80.89%	(26)
phenols	Cyclodextrin auxiliary solid extract	Methanol, cyclodextrin	88.63%	(27)
Basic polysaccharide	Alkali extraction	NaOH	79.68%	(28)
cAMP	Ultra high pressure extraction	H_2_O	1223.2ug/g	(29)
Flavonoid glycosides	Ultrasound-assisted extraction	Ethanol	50%	(30)
cAMP	Chitosan molecularly imprinted polymer	Chitosan	22.42ug/mg	(31)
Flavonoids	Ultrasound-assisted extraction	Macroporous resin	80.21mg/g	(32)
Jujube polysaccharide	Ultrasound-assisted heating extraction	Ethanol, acetone mixed with organic solvent	1.97%	(33)
GABA	Enzymatic degradation fermentation	Lactobacillus brevis	150.31ug/g	(34)
Acid polysaccharide	Alkaline extraction	NaOH	12.5%	(28)
polysaccharide	Hot water extraction	H_2_O	3.82%	(35)

## 4 Active components

Jujube contains complex chemical components and has potent biological activity. These components mainly include phenols, flavonoids, polysaccharides, triterpenoids, alkaloids, saponins, dietary fiber and other nutrients (12). Among these components, anti-cancer effects have been reported, and the main mechanisms that have been described are phenols, flavonoids, triterpenoids and saponins. In particular, the biological activities and pharmacological effects of triterpenoids (ursolic acid type, oleanane type, and lupine type) (36) have been studied in detail and adequately. As shown in **Table 2**.

**Table 2:** Bioactive components of jujube.

Indigent	Classification	Content(mg/g)	The molecular weight	Re.
Taxusin	Flavonoids	470.82 ± 8.21	504.61	(37)
Rutin	Flavonoids	66.88 ± 1.58	610.52	(24)
Unsaturated fatty acids	esters	1.66∼3.98	ND	(38)
Cellulose nanocrystals	cellulose	100	ND	(39)
BJP-4	polysaccharide	ND	1.24×105	(40)
TFC	Flavonoids	6.53 ± 0.07	ND	(25)
JPF	Flavonoids	321.6621	ND	(32)
cAMP	Adenosine	30∼160	329.21	(41)
D-Mannose	carbohydrate	35.26	180.16	(42)
Protocatechuic acid	phenol	31.54 ± 0.81	154.12	(43)
Kaempferol	Flavonoids	1.94 ± 0.018	286.24	(24)
Caffeic acid	phenol	30.84 ± 3.96	180.16	(44)
Myristic acid	Fatty acids	0.91 ± 0.01	228.37	(45)
Proanthocyanidins B2	phenol	0.273 ± 0.004	578.52	(46)
Betulinic acid	Triterpene	127000 ± 300	456.71	(47)
Oleanolic acid	Triterpene	1.8	456.71	(48)
Ent-epicatechinoceanothic acid A	Cetacean wax triterpenoids	ND	739.38	(49)

### 4.1 Phenols

Jujube contains a high content of phenolic substances, which are the main compounds (50). The extraction of active ingredients from natural plants in a safe, green, and efficient manner is highly popular. Shiva Mosadegh Manshadi et al. (51) analyzed the total flavonoids and total phenolics in red jujube using water, alcohol, and ethyl acetate extraction methods. They also determined the total phenolic content in red jujube, discussing its antiproliferative effect on acute leukemia. The presence of phenolic substances further elucidates the mechanism by which jujube treats anemia.

### 4.2 Flavonoids

Jujube is rich in various types of flavonoids. Gao et al. (52)detected the content of flavonoids in different jujube, and reported the content of 11 flavonoids. Spinosin, whose chemical formula is C_28_H_32_O_15_, is a natural flavone C-glycoside, isolated from the seeds of the jujube variety (53). At present, it has been shown that it has sedative, hypnotic and anti-anxiety pharmacological effects. In addition to this, Jianping Chen et al. (15) reported that jujube flavonoids stimulate Erythropoietin (EPO) expression. Hamid Zare et al. (52) designed a jujube syrup and proved that it had the effect of treating chronic numbness. After the analysis of its components, it was determined that the flavonoid components of jujube played a major role in its medicinal effect. The discovery of these flavonoids further expands the rich pharmacological effects of jujube. Jujube is rich in many kinds of flavonoids, which explains the diversification of its pharmacological action.

### 4.3 Triterpenes

The triterpenoids contained in jujube are mainly pentacyclic triterpenoids, which are also the bioactive markers of jujube stipulated in the Pharmacopoeia of the People’s Republic of China. At present, more than 120 kinds of triterpenoids have been proven in jujube (36). Therefore, most pharmacological studies of jujube against cancers focus on its triterpene components. The main triterpenoids reported in the literature include ursolic acid, oleanolic acid, betulinic acid, lupane, etc. Bear acid ursolic acid from primary metabolites, and the difference between it and bear fruit acid is more than a ketone group C-3 position, so it’s stronger anticancer (54). Pierluigi Plastina et al. (55) also reported the cytotoxic effect of triterpenoids from jujube on human breast cancer cells, although they did not isolate the specific triterpenoids extracted.

### 4.4 Polysaccharides

Jujube contains a high amount of polysaccharides, contributing to its sweet taste. The polysaccharides in jujube have been reported in several literatures, and their extraction, isolation and pharmacological effects have been studied. Ji et al. extracted polysaccharide fractions from jujube and obtained three kinds of jujube polysaccharides, which were discovered to have antioxidant effects (56). Jiao et al. identified four types of plant polysaccharides in jujube during their study, all of which exhibit antioxidant and anti-cancer properties (57). Xu et al. isolated and purified a deproteinized polysaccharide (DP) from jujube, which was mainly composed of two parts with average molecular weights of 143,108 and 67,633kDa, respectively. It comprised rhamnose, arabinose, xylose, mannose, glucose, and galactose in varying molar ratios. Based on this, its immunomodulatory effect was further studied (58).

### 4.5 Others

The composition of jujube is highly diverse, particularly in its bioactive ingredients, predominantly in the form of glycosides, notably flavonoid glycosides and protein glycosides, which are the primary active compounds against cancers. Jujube contains flavonoid glycosides named jujube glycosides (A-F). Jujube A and jujube B have been clearly reported. Ilandarage Menu Neelaka Molagoda, et al., studied 5 kinds of flavonoid glycosides contained in jujube, including jujube A and jujube B, and showed that these substances had obvious effects on preventing excessive melanin pigmentation (59). Additionally, other components of the jujube plant also exhibit anti-cancer effects, although few of these have been purified. Initially, these components are often extracted using organic solvents to assess their anti-cancer effects. He structure of the jujube glycosides are almost with polysaccharide by glycosidic bond together is the flavonoid glycoside is not a single flavonoids. Additionally, other components of the jujube plant also exhibit anti-cancer effects, although few of these have been purified. Initially, these components are often extracted using organic solvents to assess their anti-cancer effects. Raghuram Kandimalla et al. conducted coarse extraction fractionation of jujube root bark, revealing the anticancer effect of silymarin and its significant role in restoring liver function markers (AST, ALT, ALP, LDH, SOD, and CAT) (60). The anti-cancer mechanism of jujube may also be related to the synergistic action of several chemical components.

## 5 Mechanisms of anticancer

**Figure 4:**
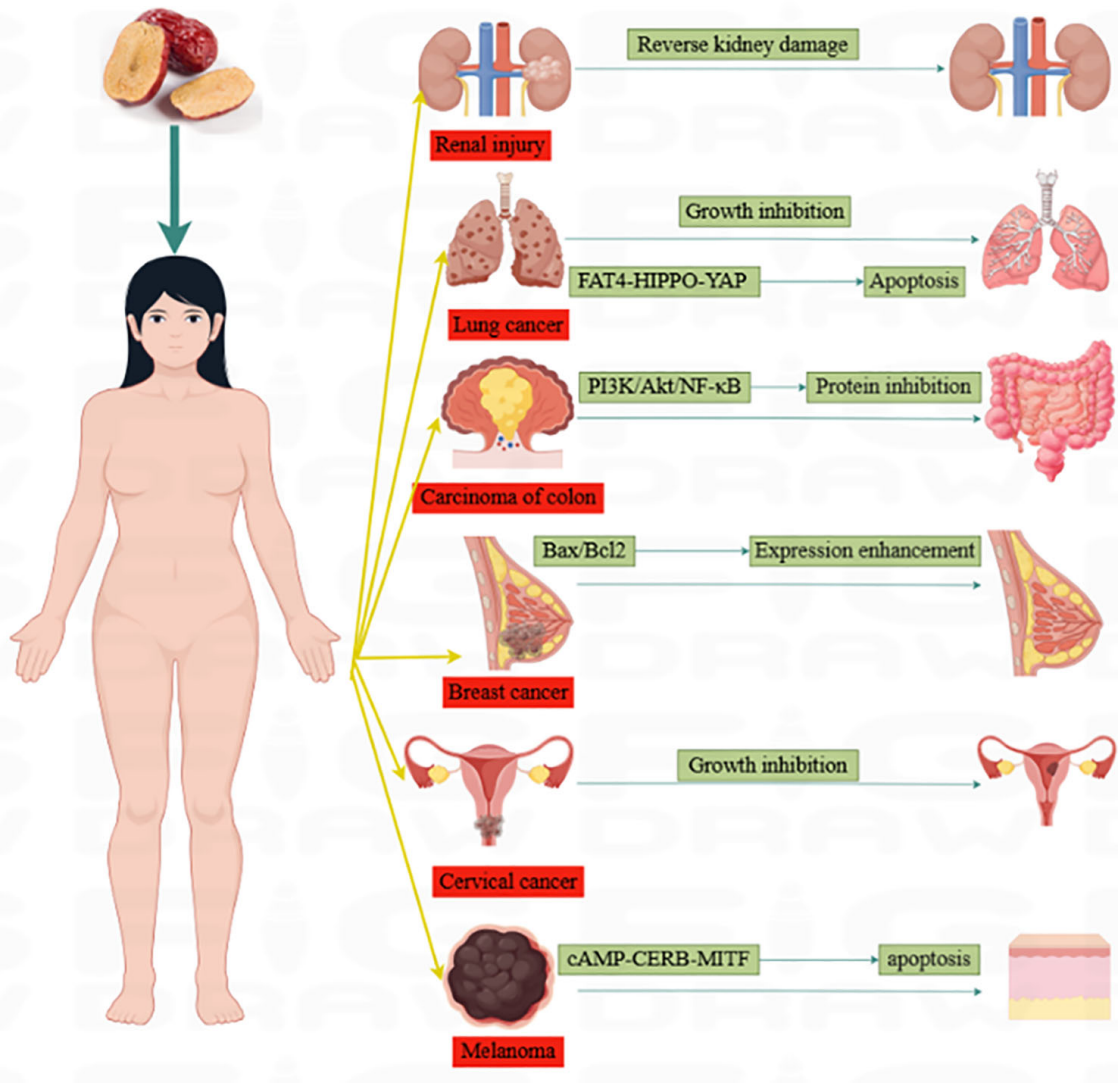
The mechanism diagram of jujube in treating cancer.

Jujube plays an important role in cancer treatment. Many scientists have conducted a complete study on the mechanism of jujube in the treatment of cancer. They found that jujube had positive effects on anti-inflammation and anti-oxidation, inhibition of cell proliferation, promotion of apoptosis, cell cycle inhibition, and DNA damage. **Figure 4**, **Table 3** represent some of the anti-cancer mechanisms of jujube.

**Table 3:** Anti-cancer mechanism of jujube.

Mechanism	Types	Pathway	IC50	Ingredient	Re.
Inhibition of protein expression	Colorectal cancer	PI3K/Akt/NF-κB pathway	14.16ug/mL	Fat-soluble containing triterpenoids	(61)
Enrich CD8-T	Colon cancer	Anti-inflammatory	800mg/Kg	Jujube powder	(62)
Anti-proliferation, apoptosis	Breast cancer	Antiestrogen receptor	14.42ug/mL	Jujube extract	(55)
Anti-proliferation apoptosis	Breast cancer, cervical cancer	enhanced Bax expression and decreased Bcl2	3mg/mL	Jujube aqueous extract	(63)
Anti-proliferation	Acute leukemia	increase Caspase-3 and decrease Bax and Bcl2	8.719mg/mL	Ethyl acetate extract of jujube	(51)
Apoptosis	Liver cancer	Damage DNA	ND	Jujube honey	(64)
Invasion migration	Liver cancer	Down-regulate Bcl-2; overexpress Caspase-3/9	2mg/mL	polysaccharide	(65)
Apoptosis	Cervical cancer	Anti-proliferation in a dose-dependent	164.6ug/mL	Jujube seed polysaccharide	(33)
Immunity regulation	Adenocarcinoma of the colon in mice	Enhanced cytotoxic CD8T cell in cancer-infiltrating	5mg/mL	Ultra-fine jujube powder	(66)
Immune activation	Colorectal cancer	Stimulate the activity of immune cell RAW264.7 to induce apoptosis;	ND	Jujube polysaccharide	(19)
Apoptosis	Jurkat leukemia	Anti-apoptosis in T- cells	0.3ug/mL	Jujube seed extract	(67)
Apoptosis	kidney injury	reduce cell autophagy; activate AMPK/mTOR signaling pathway	200ug/mL	3-dehydroceanothetric acid 2-methyl ester (3DC2ME) from jujube roots	(68)
Apoptosis	A549, PC-3, MDA-MB-231	P38-MAPK pathway	ND	terpene	(69)
Apoptosis	leukemia	lysis caspase-3	16.2mg/mL	3-O-trans-p-coumaryl-alfitoic acid (3OTPCA)	(70)
Reduced melanin production	Melanoma	Inhibition of the cAMP-CERB-MITF axis	20ug/mL	Flavonoid glycosides	(59)
Necrotizing apoptosis	Acute leukemia	RIPK1/RIPK3/MLKL pathway	ND	JU-B	(71)
Antiangiogenesis	Colorectal cancer mouse transplantation	Inhibition of MAPK pathway	100ug/mL	JU-B	(72)
Anti-phosphorylation, antiproliferation	Cancer transplantation in mice	The PI3K/Akt and MAPK/ERK signaling pathway	60umol/L	JU-B	(73)
Autophagy	Breast cancer	AMPK signaling pathway	50ug/mL	JU-B	(74)
Promoting aging	Lung cancer	FAT4-HIPPO-YAP signaling	10ug/mL	JU-A	(75)
programmed necrosis	Liver cancer	Inhibition of ROS	0.4mg/mL	Jujube polyphenol	(76)

### 5.1 Anti-inflammation and anti-oxidation

Jujube contains bioactive components such as flavonoids and flavonoid glycosides, including quercetin, which have anti-inflammatory and anti-oxidative effects. The production of inflammation is a defense mechanism produced by the body in order to restore the original homeostasis of the body when it is stimulated by pathogens, foreign substances, bacteria, etc. However, when the inflammatory response is strong, it may cause damage to some organs and impair body function (77). Cytokines associated with inflammation mainly include interleukin-6 (IL-6), interleukin-8 (IL8), cyclooxygenase-2 (COX-2), and cancer necrosis factor-α (TNF-α). Cancers, both during their development and throughout treatment, can induce inflammation and cause significant harm to the body. This is one of the main reasons the cancer cells, in the process of growth, will produce certain secretions of proinflammatory factors as well as certain chemokines induced inflammation. In the process of treatment, the use of radiation and chemotherapy drugs may have a poisonous effect on the cells caused by normal cell death induced inflammation. Wang et al. (78) detected that jujube had certain anti-inflammatory effects and briefly described its mechanism, which was mainly related to the inhibition of MAPK pathway and NF-kB. The production of a large number of free radicals is one of the characteristic phenotypes of cancers. These free radicals mainly include DPPH, ROS, NO, etc. Under the stimulation of these free radicals, it is easy to produce oxidative stress and cause damage to cells. Cheng et al. (79)discovered that jujube could remove hydroxyl free radicals and peroxides produced by hepatocytes in mice with chronic liver injury when studying jujube.

### 5.2 Inhibition of proliferation

One of the common reasons that aggressive cancers are prone to metastasis is because of the high proliferation of cancer cells. Cancer cell proliferation is often related to proliferating cell nuclear antigen (pCNA), which is involved in the important metabolism of cancer cells, such as cell survival, energy metabolism, glycolysis (80). Rapid cell proliferation is one of the characteristics of cancer cells. Most of the current common anti-cancer drugs have direct or indirect inhibitory effects on the proliferation of cancer cells. Usually, we observe the inhibition of cancer cell proliferation mainly by cell viability assay, CCK8 assay, MTT assay, etc., and observe the cell status by microscope to determine whether the cancer cells appear atrophy and whether the adherent cells float. Pierluigi Plastina et al. (81) extracted the active ingredients of jujube fruit and evaluated their effect on the proliferation of MCF-7 breast cancer cells using the MTT assay. The study found that these extracts significantly inhibited cell viability in breast cancer cells. Mohammad Reza Abedini et al. (78) obtained a jujube water extract and incubated it with OV2008 cervical cancer cells and MCF-7 breast cancer cells. Observations of cell morphology and MTT assays of cell viability revealed a significant antiproliferative effect on cancer cells within 24 to 72 hours, with the cancer cells exhibiting atrophy, bubbling, and other morphological changes.

### 5.3 Promotion of apoptosis

Apoptosis, also known as programmed cell death (82), is a normal cell death mode. By promoting the natural death of cancer cells in certain ways, it can ensure the anti-cancer effect while avoiding a large number of toxic side effects brought by radiotherapy and chemotherapy. At present, there are more and more studies on how to promote programmed cell death in cancer cells. The common apoptotic pathways of cancer cells include the mitochondrial pathway, caspase dependent pathway and so on. Caspase-3 is a key enzyme in the apoptosis pathway, and an increase in its activity indicates an increase in cell apoptosis. Merve Nur Ataş et al. (83) studied the mechanism of betulinic acid in promoting cell apoptosis. Betulinic acid is one of the hallmark components of jujube. In this study, betulinic acid was found to increase the enrichment of apoptotic bodies in renal cancer cells. The expression of AKT-1 and mTOR, two genes that induce cell survival, was significantly reduced in response to the treatment. Zhang et al. (84) also conducted similar studies, and they proposed that betulinic acid can inhibit cell apoptosis by upregulating caspase-3, and in addition, it can promote autophagy dependent apoptosis of bladder cancer cells through Bmi-1/ROS/AMPK-mTOR-ULK1 signaling axis. Wu et al. (33) extracted polysaccharides contained in jujube seeds and verified that their anti-cancer effect was achieved through cell apoptosis after a series of structural characterization, extraction, and separation.

### 5.4 Cell aging

The four phenotypes of cellular senescence recognized by the International Society of Cellular Senescence include: cell cycle exit, macromolecular damage, secretory phenotype and Metabolic disorders (85). Modern studies on the anti-cancer effects of jujube mostly indicate that its primary effect is to induce cancer cell senescence without affecting normal cells. This characteristic underlies the minimal toxicity and side effects associated with many natural anti-cancer agents.

#### 5.4.1 Induction of cycle arrest

The uninterrupted division of cancer cells is one of the main reasons for its difficulty in treatment. Disrupting cancer cell growth arrest or inducing cancer cell death by targeting genes or proteins involved in the cell cycle is a critical strategy in cancer treatment. There are many mechanisms that can promote the cycle arrest of cancer cells, and we briefly review them. The p53 transcription factor is a well-known protein that inhibits cancer cells in the G1 phase. It can interact with the anti-apoptotic proteins of the BCL2 family, leading to their inactivation while allowing other pro-apoptotic proteins to function effectively (86). Upregulation of P21 level increases the level of cell cycle dependent kinase CDK family cut, which can cause the stagnation of cells in S phase. Influence on cell cycle proteins can also make the cancer cell cycle stagnant. The content of ursolic acid in jujube is high, which has the effect of inducing cell arrest in cancer cells, thus exerting its anti-cancer effect.

The mechanism by which ursolic acid induces cancer cell cycle arrest involves its entry into NTERA-2 and NCCIT cells, leading to increased expression of p21. This process inhibits cyclins E and D1, as well as CDK proteins, thereby extending the duration of cancer cell cycle arrest (87). Wang et al. (75) discovered that JUA is a novel activator of FAT4, a cancer suppressor in lung cancer, which causes cell cycle arrest primarily by activating the FAT4-Hippo signaling pathway and promoting YAP nuclear translocation.

#### 5.4.2 Damage DNA

Cells rely on DNA repair and DNA damage to remove mutations or genetic diseases to maintain the stability of the body and heredity, and these two responses are collectively referred to as DNA damage response (DDR). The DDR reaction in cancer cells is highly fragile and easily compromised, leading to the loss of cell cycle checkpoints. Consequently, cancer cells exhibit accelerated growth and reproduction.

At present, the commonly used method to treat cancer is to activate the cancer cell cycle checkpoints such as ATM, ATR, CHK and other genes, so that cancer cells can die normally. However, ATM gene is inactivated in most cancer cells, but fortunately, ATM can be activated by ROS to phosphorylate p53 and CHK2 and other apoptotic factors, leading to cancer cell apoptosis. In addition, ATM activation can also phosphorylate the NF-kB pathway and lead to cell apoptosis, which is the classic ATM-CHK2 pathway (88). In addition, there is the ATR-CHK1 pathway. Shen et al. (69) extract and isolate the active components of triterpenoids in jujube and discover that they have cytotoxic effects on cancer cells. They also study the mechanism of apoptosis induced by them, which is that they stimulated mitochondria to produce ROS and activate ATM to phosphorylate p38-MAPK, leading to cancer cell apoptosis.

#### 5.4.3 Increase of secretion factors

The increase of secretory factors, including inflammatory factors such as IL6 and IL8, is also one of the factors causing cancer cell senescence. These secreted factors, such as MMPs, are present in a variety of signaling pathways. Cisplatin, as a commonly used drug in chemotherapy, can cause irreversible damage to normal cells in the process of reducing cancer cells, and cisplatin is extremely commonly used in the treatment of ovarian cancer and renal cancer. Long-term use of platinum drugs will cause serious toxic effects on normal cells, causing irreversible kidney damage and ovarian damage. Li et al. (68) extracted the active ingredients of Jujube root part and discovered that it contains unique triterpenoids, which can affect some secreted factors to activate the 5 ‘AMPK factor in the mTOR dependent signaling pathway to inhibit the damage of cisplatin to normal renal epithelial cells.

#### 5.4.4 Cell metabolism disorders

The difference between cancer cells and normal cell metabolism is that cancer cells can obtain the energy needed by lipid metabolism, biological ingredients, etc. In addition, glycolysis and cholesterol metabolism in cancer cell metabolism also play an indispensable role. By changing the cancer cell metabolism, glycolysis metabolic processes, such as necrotizing apoptosis, can promote cell apoptosis and even affect the treatment of cancers. Wang et al (89) discovered in the process of studying ursolic acid (one of the main triterpene acids contained in jujube) that ursolic acid can activate the enzyme Caveolin-1 (Cav-1), which can well inhibit some rate-limiting enzymes in the glycolysis process, thus affecting the glycolysis process of cancer cells and causing apoptosis. In addition, affecting the respiration of mitochondria can also achieve this therapeutic purpose.

### 5.5 Cell autophagy

Autophagy is usually caused by the release of autophagosomes from lysosomes under the influence of certain factors, which is a process of cell lysis. Normally, normal cells will send signals to lysosomes under the control of DNA after growth to a certain cycle or lesions, leading to autophagy in the cell itself. Under the action of kinase ULK1, the new membrane of autophagosomes produced by the endoplasmic reticulum begins to lipidate and increase the membrane area, which also means the start of autophagy. Under the regulation of LC3 enzyme and ATG8 protein, autophagosomes will perform selective phagocytosis, so as to keep cells in a healthy state. In the process of cancer treatment, we can regulate autophagy and remove specific factors to promote cancer inhibition (90). DitteLFogde et al. (91) discovered a possible mechanism when studying ursolic acid. Ursolic acid can change the pH value of the lysosome in cancer cells, thereby changing the permeability of the lysosomal membrane, causing errors in cell autophagy and resulting in cancer inhibition. Oleanolic acid is also one of the index components of jujube. Iva Potočnjak et al. (92) discovered that oleanolic acid induced autophagy in colon cancer cells by increasing the expression of LC3B and reducing the expression of mitochondrial outer membrane 20 translocase.

### 5.6 Immunoregulation

Immune regulation is mainly manifested in the regulation of the body level and the regulation of the cell level. Through immune regulation, the function of the immune system is enhanced, and the proliferation of immune cells is promoted to complete the killing effect on cancer cells. Ruan et al. (93) reviewed jujube polysaccharides and their main pharmacological effects, and showed that JUBP-1, a polysaccharide extracted and isolated from jujube, had a good immune enhancement effect, which was mainly manifested as the induction of spleen cell proliferation. Interestingly, JU-4, another polysaccharide contained in jujube, is known to act as an immunomodulatory factor with a direct ameliorating effect on the immune system. Sandeep Kumar Dash et al. (94) discovered that when the birch fatty acid, self-assembly birch fatty acid regulation on IGg can stimulate the body’s humoral and cellular immunity, stimulate the secretion of macrophage cancer necrosis factor and anti-inflammatory factor has a cytotoxic effect on cancer.

## 6 Attenuation and synergism

Cancer treatment often affects patients more than the tumor itself. At present, how to reduce the toxic effects of cancer treatment to patients and enhance the efficacy of chemotherapy drugs is also gradually becoming popular. Jujube can not only treat cancers, but also has a good easing effect on some toxic side effects caused by radiotherapy and chemotherapy treatment. At the same time, it can also enhance the therapeutic effect of cancer.

### 6.1 Attenuation effect

#### 6.1.1 Liver and kidney damage

Cisplatin is one of the commonly used chemotherapy drugs for the treatment of cancers. Long-term administration of cisplatin can cause irreversible liver and kidney damage. Long-term exposure to cisplatin can cause autophagy of renal epithelial cells and lead to apoptosis of renal epithelial cells, which is related to the inhibition of AMPK/mTOR apoptosis pathway. Li et al. (68) extracted the unique triterpene 3DC2ME from jujube root, and discovered that it could inhibit the above pathways and protect renal epithelial cells. Liu et al. discovered that jujube polysaccharide had a good protective effect on liver injury in mice induced by a variety of drugs, including CCL4 and APAP (95). For most cancer patients, long-term use of anti-cancer drugs causes an irreversible injury to liver and kidney function. Jujube’s protection of liver and kidney, can reduce the adverse reaction.

#### 6.1.2 Burden of the heart

One of the common characteristics of cancer patients is the excessive burden on the heart, especially since the long-term use of antineoplastic drugs will produce significant cardiac toxicity. Dong et al. (96) have discovered that JU-B can nourish the heart and attenuate cardiotoxicity. Reza Mohebbati et al. (97) discovered that the aqueous solution of jujube extract can promote coronary vasodilatation by increasing NO, thereby increasing cardiac blood flow and reducing blood pressure, which is an effective and beneficial effect to reduce the burden on the heart for patients receiving long-term anti-cancer treatment.

#### 6.1.3 Anxiety

The negative impact of cancers on patients is not limited to their impact on physical function. Long-term cancer patients will also suffer certain damage to the nervous system, which manifests as anxiety, depression, insomnia and drug-affected intestinal flora imbalance. Jujube kernel (Ziziphus jujube kernel) has been considered to have anti-anxiety, sedative and regulating intestinal flora in traditional Chinese medicine since ancient times (98). Liu et al. studied that jujube kernel has obvious anti-anxiety effect, and conducted a mechanism study in mouse model and discovered that its anti-anxiety effect is mainly related to GABA and 5-HT (99). Yang et al. (100) identified the prevalence of sleep disorders among cancer patients and conducted a systematic review and meta-analysis. They concluded that jujube seed can effectively ameliorate cancer-induced sleep disorders. However, the underlying mechanism remains unstudied. Bian et al. (101) investigated the effect of jujube kernel on sleep improvement using metabonomics. Their study revealed that flavonoid extracts contained in jujube kernel target multiple pathways to enhance sleep, a finding corroborated through pharmacological network analysis. Qiao et al. discovered that jujube kernels can increase the abundance of intestinal flora and improve sleep in rats by regulating the GLU/GABA-GLN metabolic cycle through the brain-gut axis (102).

#### 6.1.4 Fungal infections

Long-term chemotherapy often results in immune decline and increases susceptibility to bacterial infections. The standard treatment for bacterial infections remains the use of antibiotics, which frequently cause significant liver and kidney damage. For cancer patients, who may already suffer from impaired liver and kidney function, this presents an additional burden. Jujube, however, contains flavonoids, polysaccharides, and terpenoids, which exhibit strong antibacterial properties and may offer a beneficial alternative or complement to conventional antibiotic treatments.

Xu et al. (103) discovered that water-soluble jujube polysaccharide can inhibit the activity of a variety of oral pathogenic bacteria, thereby preventing oral pathogen infection. In addition, Miao et al. (104) discovered that the extract of jujube can resist the biofilm formation of Staphylococcus aureus, which provides strong evidence for the antibacterial effect of jujube.

### 6.2 Synergism effect

#### 6.2.1 Anti-radiation

Gamma rays are commonly used in radiotherapy to kill cancer cells. They have a powerful killing effect and also have a huge toxic effect on normal cells. Patients who have received radiotherapy for a long time have symptoms such as decreased immunity, hair loss, and decreased body function. Ujjal Das et al. (105) discovered that ferulic acid extracted from jujube had an inhibitory effect on the ROS level of lung cancer cells and liver cancer cells. Based on this, ferulic acid could improve the sensitivity of cancer cells to gamma rays, so as to achieve a potent cytotoxic effect that does not affect the normal function of the body of human body.

#### 6.2.2 Intestinal flora

Colorectal cancer often leads to intestinal flora disorders, and the intestinal microbiota plays a crucial role in the development and progression of colorectal cancer. Ji et al. (106) discovered that jujube polysaccharides can significantly reduce the abundance of Firmicutes and Bacteroidetes, indicating that jujube polysaccharides may serve as prebiotics. This suggests their potential in regulating intestinal microbiota to improve and prevent colorectal cancer. Most studies have shown that intestinal flora, as digestive microorganisms, affect the occurrence and prevention of many human diseases. The improvement of the gut microbiota further clarified that jujube can significantly improve cancer treatment and prognosis during cancer treatment.

#### 6.2.3 Cancer-related anemia

Cancer-related anemia is a very common complication of cancer. There are many reasons for its occurrence, such as bone marrow transplantation caused by radiotherapy and chemotherapy, Inflammation caused by bleeding, Bone marrow necrosis caused by cytoma, myeloma, etc. Abnormal immune system responses, such as those caused by hemolysis, can lead to anemia. Currently, the primary treatments for cancer-related anemia include blood transfusions and erythropoietin stimulants, both of which can cause significant harm to the body. Xu et al. (107) discovered that a unique carbon point, J-CD, derived from jujube can specifically stimulate the self-renewal of red blood cell precursors, thereby promoting red blood cell production without affecting cancer proliferation and metastasis. This finding holds promise for treating cancer-related anemia, potentially reducing the harm to the bodies of cancer patients.

#### 6.2.4 Combined administration

Doxorubicin and adriamycin are commonly used chemotherapy drugs in the treatment of cancer. However, these drugs have significant side effects, the most serious being the induction of apoptosis in normal cells and the triggering of severe inflammatory responses. Sandeep Kumar Dash et al. (108) discovered in their study that betulinic acid extracted from the bark of jujube could protect peripheral lymphocytes from oxidative stress induced by adriamycin, and they also verified this effect by Western Blot and other experiments. These results indicated that betulinic acid could alleviate the cytotoxic effect of chemotherapeutic drugs on normal cells. This is also helpful for patients with long-term chemotherapy.

#### 6.2.5 Combined with tea

In China, the practice of drinking jujube tea has a long history, but few studies have explored the combined effects of jujube and tea. Huang et al. (107) found that while jujube alone had a lower selective killing effect on hepatoma cell line HepG2, the jujube green tea extract exhibited a significantly higher selective killing effect on these cancer cells without adversely affecting normal liver cells. The mechanism is likely related to the growth inhibition of cancer cells by the jujube tea extract. Additionally, they discovered that the jujube tea extract had no effect on the natural apoptosis of cells, but specifically targeted the G1 phase of cancer cells. The mechanism of this effect differed from that of jujube extract alone. These findings are promising for further research on the benefits of tea consumption (109).

## 7 Conclusion

Jujube, as a food, medicine and fruit plant, is very popular in the world. This review summarizes the extraction and separation of the active ingredients of jujube, delineates their anti-cancer effects across various cancer types, and explores their functions in mitigating some adverse reactions associated with cancer treatment. By summarizing the previous literature, it is discovered that jujube can treat a variety of cancers, such as lung cancer, blood cancer, breast cancer, colorectal cancer, etc. The treatment process involves a variety of mechanisms, including promoting cell apoptosis, immune regulation, promoting autophagy, etc. While treating cancers, it also has a positive effect on some adverse reactions caused by chemotherapy, including inflammation, liver and kidney damage, fungal infection, anxiety, sleep disorders, etc. This illustrates that jujube consumption can mitigate cancer-related adverse effects and exert direct anti-cancer effects on cancer cells. The increasing recognition of the potent efficacy and minimal side effects of natural products as a novel source of antineoplastic drugs provides strong support. Further exploration of jujube’s anti-cancer effects will aid in expanding the search for anti-cancer agents to encompass everyday consumables and common dietary items.

## 8 Outlook

Currently, the medicinal application of jujube predominantly remains within traditional Chinese medicine, with limited use in Western medicine. With the rapid development of the pharmaceutical industry, a large number of new drug preparation methods, such as nanoparticle system, chitosan, microcapsules, and pellets, have been developed to rapidly and accurately deliver drugs to the disease site. However, there are limited studies on effectively integrating the active ingredients of jujube with these preparations to promote its clinical utilization. Moreover, research on the active constituents of jujube for cancer treatment is predominantly limited to animal and cell experiments, with rare reports on cancer regression in humans and patients following the administration of related preparations. Future investigations should focus on the chemical modification and structural transformation of compounds within jujube possessing anticancer properties to enhance their activity and generate targets that are more beneficial for patients with diverse cancer types.

## References

[1] Panjwani AA Li M. Recent trends in the management of depression in persons with cancer. Curr Opin Psychiatry. (2021) 34:448–59. doi: 10.1097/YCO.0000000000000727 34224469

[2] Pasquini M Biondi M. Depression in cancer patients: a critical review. Clin Pract Epidemiol Ment Health. (2007). doi: 10.1186/1745-0179-3-2 PMC1797173 17288583

[3] Roy PS Saikia BJ. Cancer and cure: A critical analysis. Indian J Cancer. (2016) 53:441–2. doi: 10.4103/0019-509X.200658 28244479

[4] Zaimy MA Saffarzadeh N Mohammadi A Pourghadamyari H Izadi P Sarli A. New methods in the diagnosis of cancer and gene therapy of cancer based on nanoparticles. Cancer Gene Ther. (2017) 24:233–43. doi: 10.1038/cgt.2017.16 28574057

[5] Naeem A Hu P Yang M Zhang J Liu Y Zhu W. Natural products as anticancer agents: current status and future perspectives. Molecules. (2022) 27. doi: 10.3390/molecules27238367 PMC9737905 36500466

[6] Qi L Zhong F Chen Y Mao S Yan Z Ma Y. An integrated spectroscopic strategy to trace the geographical origins of emblic medicines: Application for the quality assessment of natural medicines. J Pharm Anal. (2020) 10:356–64. doi: 10.1016/j.jpha.2019.12.004 PMC7474118 32923010

[7] Shen H-S Wen S-H. Effect of early use of Chinese herbal products on mortality rate in patients with lung cancer. J Ethnopharmacol. (2018) 211:1–8. doi: 10.1016/j.jep.2017.09.025 28942131

[8] Qiang X Xia T Geng B Zhao M Li X Zheng Y. Bioactive components of lycium barbarum and deep-processing fermentation products. Molecules. (2023) 28. doi: 10.3390/molecules28248044 PMC10745962 38138534

[9] Jiang M Zhao S Yang S Lin X He X Wei X. An “essential herbal medicine”-licorice: A review of phytochemicals and its effects in combination preparations. J Ethnopharmacol. (2020) 249:112439. doi: 10.1016/j.jep.2019.112439 31811935

[10] Zhou S Feng D Zhou Y Duan H Jiang Y Yan W. Analysis of the active ingredients and health applications of cistanche. Front Nutr. (2023) 10:1101182. doi: 10.3389/fnut.2023.1101182 36992906 PMC10042234

[11] Zou L Li H Ding X Liu Z He D Kowah JAH. A review of the application of spectroscopy to flavonoids from medicine and food homology materials. Molecules. (2022) 27. doi: 10.3390/molecules27227766 PMC9696260 36431869

[12] Lu Y Bao T Mo J Ni J Chen W. Research advances in bioactive components and health benefits of jujube (Ziziphus jujuba Mill.) fruit. J Zhejiang Univ Sci B. (2021) 22:431–49. doi: 10.1631/jzus.B2000594 PMC8214949 34128368

[13] Gao Q-H Wu C-S Wang M. The jujube (Ziziphus jujuba Mill.) fruit: a review of current knowledge of fruit composition and health benefits. J Agric Food Chem. (2013) 61:3351–63. doi: 10.1021/jf4007032 23480594

[14] Ji X Peng Q Yuan Y Shen J Xie X Wang M. Isolation, structures and bioactivities of the polysaccharides from jujube fruit (Ziziphus jujuba Mill.): A review. Food Chem. (2017) 227:349–57. doi: 10.1016/j.foodchem.2017.01.074 28274443

[15] Chen J Tsim KWK. A review of edible jujube, the ziziphus jujuba fruit: A heath food supplement for anemia prevalence. Front Pharmacol. (2020) 11:593655. doi: 10.3389/fphar.2020.593655 33324222 PMC7726020

[16] Wu Z Zhang S Liu L Wang L Ban Z. The Grade of Dried Jujube (Ziziphus jujuba Mill. cv. Junzao) Affects Its Quality Attributes, Antioxidant Activity, and Volatile Aroma Components. Foods. (2023) 12. doi: 10.3390/foods12050989 PMC10000541 36900506

[17] Hoseinifar SH Khodadadian Zou H Van Doan H Harikrishnan R Yousefi M Paknejad H. Can dietary jujube (Ziziphus jujuba Mill.) fruit extract alter cutaneous mucosal immunity, immune related genes expression in skin and growth performance of common carp (Cyprinus carpio)? Fish Shellfish Immunol. (2019) 94:705–10. doi: 10.1016/j.fsi.2019.09.016 31505247

[18] Chen J Du CYQ Lam KYC Zhang WL Lam CTW Yan AL. The standardized extract of Ziziphus jujuba fruit (jujube) regulates pro-inflammatory cytokine expression in cultured murine macrophages: suppression of lipopolysaccharide-stimulated NF-κB activity. Phytother Res. (2014) 28:1527–32. doi: 10.1002/ptr.5160 24806434

[19] Liang Q Wang X Yang S Yu L Gao Q Yang X. Characterization of the antioxidative polysaccharides from Ziziphus jujube cv. Goutouzao and its tumor-inhibitory effects on human colorectal carcinoma LoVo cells *via* immunocyte activation. J Food Biochem. (2020) 44:e13462. doi: 10.1111/jfbc.13462 32954518

[20] Zeng B Qi L Wu S Liu N Wang J Nie K. Network Pharmacology Prediction and Metabolomics Validation of the Mechanism of Fructus Phyllanthi against Hyperlipidemia. J Vis Exp. (2023) 7(194). doi: 10.3791/65071 37092814

[21] Wang J Dong J Zhong F Wu S An G Liao W. Microbiome-Metabolome Analysis Insight into the Effects of the Extract of Phyllanthus emblica L. @ on High-Fat Diet-Induced Hyperlipidemia. Metabolites. (2024) 14. doi: 10.3390/metabo14050257 PMC11123125 38786734

[22] Zhao K Qi L Li Q Wang Y Qian C Shi Z. Self-absorbing multilayer skin-like composite with Phyllostachys nigra polysaccharides promotes wound healing. Adv Composites And Hybrid Mater. (2024) 7. doi: 10.1007/s42114-024-01018-x

[23] Zou X Xiao J Chi J Zhang M Zhang R Jia X. Physicochemical properties and prebiotic activities of polysaccharides from Zizyphus jujube based on different extraction techniques. Int J Biol Macromol. (2022) 223:663–72. doi: 10.1016/j.ijbiomac.2022.11.057 36368360

[24] Liu X Liu Y Shan C Yang X Zhang Q Xu N. Effects of five extraction methods on total content, composition, and stability of flavonoids in jujube. Food Chem: X. (2022) 14:100287. doi: 10.1016/j.fochx.2022.100287 35313650 PMC8933822

[25] Zhu J Kou X Wu C Fan G Li T Dou J. Enhanced extraction of bioactive natural products using ultrasound-assisted aqueous two-phase system: Application to flavonoids extraction from jujube peels. Food Chem. (2022) 395:133530. doi: 10.1016/j.foodchem.2022.133530 35777209

[26] Patel G Patra A Abdullah S Dwivedi M. Indian jujube (Ziziphus mauritiana L.) fruit juice extraction using cellulase enzyme: Modelling and optimization of approach by ANN-GA. Appl Food Res. (2022) 2:100080. doi: 10.1016/j.afres.2022.100080

[27] Zhu Q-Y Zhang Q-Y Cao J Cao W Xu J-J Peng L-Q. Cyclodextrin-assisted liquid-solid extraction for determination of the composition of jujube fruit using ultrahigh performance liquid chromatography with electrochemical detection and quadrupole time-of-flight tandem mass spectrometry. Food Chem. (2016) 213:485–93. doi: 10.1016/j.foodchem.2016.06.115 27451208

[28] Lin X Ji X Wang M Yin S Peng Q. An alkali-extracted polysaccharide from Zizyphus jujuba cv. Muzao: Structural characterizations and antioxidant activities. Int J Biol Macromol. (2019) 136:607–15. doi: 10.1016/j.ijbiomac.2019.06.117 31220502

[29] Sang C Bai Q Feng X Wu C Liu Y Gao Z. Optimized extraction of cAMP from jujube by ultra-high pressure technology and the anti-allergic effect for peanut allergy mouse. Front Nutr. (2022) 9:862900. doi: 10.3389/fnut.2022.862900 35719140 PMC9199853

[30] Liu J Zhang Y Zhang M Wang Q Xie J. Ultrasonic-assisted extraction of swertisin from sour Jujube seed and comprehensive revelation of its antioxidant activity. J Food Biochem. (2022) 46:e14433. doi: 10.1111/jfbc.14433 36198041

[31] Li F Li X Su J Li Y He X Chen L. Hydrophilic molecularly imprinted polymers functionalized magnetic carbon nanotubes for selective extraction of cyclic adenosine monophosphate from winter jujube. J Sep Sci. (2021) 44:2131–42. doi: 10.1002/jssc.202001095 33721391

[32] Shen D Labreche F Wu C Fan G Li T Dou J. Ultrasound-assisted adsorption/desorption of jujube peel flavonoids using macroporous resins. Food Chem. (2022) 368:130800. doi: 10.1016/j.foodchem.2021.130800 34403997

[33] Wu Z Li H Wang Y Yang D Tan H Zhan Y. Optimization extraction, structural features and antitumor activity of polysaccharides from Z. jujuba cv. Ruoqiangzao seeds. Int J Biol Macromol. (2019) 135:1151–61. doi: 10.1016/j.ijbiomac.2019.06.020 31173825

[34] Bae GY Ahn Y Hong K-B Jung E-J Suh HJ Jo K. Sleep-Enhancing Effect of Water Extract from Jujube (Zizyphus jujuba Mill.) Seeds Fermented by Lactobacillus brevis L32. Foods. (2023) 12. doi: 10.3390/foods12152864 PMC10417159 37569133

[35] Ji X Yan Y Hou C Shi M Liu Y. Structural characterization of a galacturonic acid-rich polysaccharide from Ziziphus Jujuba cv. Muzao. Int J Biol Macromol. (2020) 147:844–52. doi: 10.1016/j.ijbiomac.2019.09.244 31743713

[36] Pan F Zhao X Liu F Luo Z Chen S Liu Z. Triterpenoids in jujube: A review of composition, content diversity, pharmacological effects, synthetic pathway, and variation during domestication. Plants (Basel). (2023) 12. doi: 10.3390/plants12071501 PMC10096698 37050126

[37] Nuralın L. Investigation of ziziphus jujube seeds as a new source of taxifolin and silibinin with three different extraction methods. Microchemical J. (2024) 198:110137. doi: 10.1016/j.microc.2024.110137

[38] Fu L Yang J Shang H Song J. Changes of characteristic sugar, fatty acid, organic acid and amino acid in jujubes at different dry mature stages. J Food Composition Anal. (2021) 104:104104. doi: 10.1016/j.jfca.2021.104104

[39] Maheri H Hashemzadeh F Shakibapour N Kamelniya E Malaekeh-Nikouei B Mokaberi P. Glucokinase activity enhancement by cellulose nanocrystals isolated from jujube seed: A novel perspective for type II diabetes mellitus treatment (*In vitro*). J Mol Structure. (2022) 1269:133803. doi: 10.1016/j.molstruc.2022.133803

[40] Liu C Qiu Z Gu D Wang F Zhang R. A novel anti-inflammatory polysaccharide from blackened jujube: Structural features and protective effect on dextran sulfate sodium-induced colitic mice. Food Chem. (2023) 405:134869. doi: 10.1016/j.foodchem.2022.134869

[41] Jiang T He F Han S Chen C Zhang Y Che H. Characterization of cAMP as an anti-allergic functional factor in Chinese jujube (Ziziphus jujuba Mill.). J Funct Foods. (2019) 60:103414. doi: 10.1016/j.jff.2019.06.016

[42] Jin H-F Shi Y Shi M-Z Cao J Ye L-H. Ionic liquid-assisted aqueous two-phase system for the enrichment of multiple monosaccharides from jujube. Ind Crops Products. (2023) 204:117392. doi: 10.1016/j.indcrop.2023.117392

[43] Dou J-F Wu C-E Fan G-J Li T-T Li X-J Zhou D-D. Insights into the pigment and non-pigment phenolic profile of polyphenol extracts of jujube peel and their antioxidant and lipid-lowering activities. Food Biosci. (2023) 52:102493. doi: 10.1016/j.fbio.2023.102493

[44] Liu Y Liao Y Guo M Zhang W Sang Y Wang H. Comparative elucidation of bioactive and volatile components in dry mature jujube fruit (Ziziphus jujuba Mill.) subjected to different drying methods. Food Chem: X. (2022) 14:100311. doi: 10.1016/j.fochx.2022.100311 35492255 PMC9043666

[45] Reche J Almansa MS Hernández F Carbonell-BarraChina ÁA Legua P Amorós A. Fatty acid profile of peel and pulp of Spanish jujube (Ziziphus jujuba Mill.) fruit. Food Chem. (2019) 295:247–53. doi: 10.1016/j.foodchem.2019.05.147 31174755

[46] Wojdyło A Carbonell-BarraChina ÁA Legua P Hernández F. Phenolic composition, ascorbic acid content, and antioxidant capacity of Spanish jujube (Ziziphus jujube Mill.) fruits. Food Chem. (2016) 201:307–14. doi: 10.1016/j.foodchem.2016.01.090 26868581

[47] Guo S Duan J-A Qian D Tang Y Wu D Su S. Content variations of triterpenic acid, nucleoside, nucleobase, and sugar in jujube (Ziziphus jujuba) fruit during ripening. Food Chem. (2015) 167:468–74. doi: 10.1016/j.foodchem.2014.07.013 25149013

[48] Kawabata K Kitamura K Irie K Naruse S Matsuura T Uemae T. Triterpenoids isolated from ziziphus jujuba enhance glucose uptake activity in skeletal muscle cells. J Nutr Sci Vitaminol (Tokyo). (2017) 63:193–9. doi: 10.3177/jnsv.63.193 28757534

[49] Kang KB Kim HW Kim JW Oh WK Kim J Sung SH. Catechin-bound ceanothane-type triterpenoid derivatives from the roots of zizyphus jujuba. J Nat Prod. (2017) 80:1048–54. doi: 10.1021/acs.jnatprod.6b01103 28257196

[50] Abdoul-Azize S. Potential benefits of jujube (Zizyphus lotus L.) bioactive compounds for nutrition and health. J Nutr Metab. (2016) 2016:2867470. doi: 10.1155/2016/2867470 28053781 PMC5174181

[51] Mosadegh Manshadi S Shams Ardekani MR. Antitumor activity of ziziphus jujube fruit extracts in KG-1 and NALM-6 acute leukemia cell lines. Int J Hematol Oncol Stem Cell Res. (2023) 17:257–66. doi: 10.18502/ijhoscr.v17i4.13917 PMC10700102 38076779

[52] Gao Q-H Wu C-S Yu J-G Wang M Ma Y-J Li C-L. Textural characteristic, antioxidant activity, sugar, organic acid, and phenolic profiles of 10 promising jujube (Ziziphus jujuba Mill.) selections. J Food Sci. (2012) 77:C1218–1225. doi: 10.1111/j.1750-3841.2012.02946.x 23057538

[53] Kuang X She G Ma T Cai W Zhao J Liu B. The pharmacology, pharmacokinetics, and toxicity of spinosin: A mini review. Front Pharmacol. (2022) 13:938395. doi: 10.3389/fphar.2022.938395 36193419 PMC9525219

[54] Son J Lee SY. Therapeutic potential of ursonic acid: comparison with ursolic acid. Biomolecules. (2020) 10. doi: 10.3390/biom10111505 PMC7693102 33147723

[55] Plastina P Bonofiglio D Vizza D Fazio A Rovito D Giordano C. Identification of bioactive constituents of Ziziphus jujube fruit extracts exerting antiproliferative and apoptotic effects in human breast cancer cells. J Ethnopharmacol. (2012) 140:325–32. doi: 10.1016/j.jep.2012.01.022 22301448

[56] Ji X Hou C Yan Y Shi M Liu Y. Comparison of structural characterization and antioxidant activity of polysaccharides from jujube (Ziziphus jujuba Mill.) fruit. Int J Biol Macromol. (2020) 149:1008–18. doi: 10.1016/j.ijbiomac.2020.02.018 32032709

[57] Jiao R Liu Y Gao H Xiao J So KF. The anti-oxidant and antitumor properties of plant polysaccharides. Am J Chin Med. (2016) 44:463–88. doi: 10.1142/S0192415X16500269 27109156

[58] Hsu B-Y Kuo Y-C Chen B-H. Polysaccharide isolated from zizyphus jujuba (Hóng zăo) inhibits interleukin-2 production in jurkat T cells. J Tradit Complement Med. (2014) 4:132–5. doi: 10.4103/2225-4110.124360 PMC4003703 24860737

[59] Molagoda IMN Lee K-T Athapaththu AMGK Choi Y-H Hwang J Sim S-J. Flavonoid Glycosides from Ziziphus jujuba var. inermis (Bunge) Rehder Seeds Inhibit α-Melanocyte-Stimulating Hormone-Mediated Melanogenesis. Int J Mol Sci. (2021) 22. doi: 10.3390/ijms22147701 PMC8304508 34299326

[60] Kandimalla R Dash S Kalita S Choudhury B Malampati S Kalita K. Protective Effect of Bioactivity Guided Fractions of Ziziphus jujuba Mill. Root Bark against Hepatic Injury and Chronic Inflammation *via* Inhibiting Inflammatory Markers and Oxidative Stress. Front Pharmacol. (2016) 7:298. doi: 10.3389/fphar.2016.00298 27656145 PMC5013132

[61] Ruan J Li H Lu M Hao M Sun F Yu H. Bioactive triterpenes of jujube in the prevention of colorectal cancer and their molecular mechanism research. Phytomedicine. (2023) 110:154639. doi: 10.1016/j.phymed.2022.154639 36608502

[62] Zhuang H Jing N Wang L Jiang G Liu Z. Jujube powder enhances cyclophosphamide efficiency against murine colon cancer by enriching CD8(+) T cells while inhibiting eosinophilia. Nutrients. (2021) 13. doi: 10.3390/nu13082700 PMC8401958 34444860

[63] Abedini MR Erfanian N Nazem H Jamali S Hoshyar R. Anti-proliferative and apoptotic effects of Ziziphus Jujube on cervical and breast cancer cells. Avicenna J Phytomed. (2016) 6:142–8. PMC4877962 27222827

[64] Cheng N Zhao H Chen S He Q Cao W. Jujube honey induces apoptosis in human hepatocellular carcinoma HepG2 cell *via* DNA damage, p53 expression, and caspase activation. J Food Biochem. (2019) 43:e12998. doi: 10.1111/jfbc.12998 31373040

[65] Zhang G Liu C Zhang R. A novel acidic polysaccharide from blackened jujube: Structural features and antitumor activity *in vitro* . Front Nutr. (2022) 9:1001334. doi: 10.3389/fnut.2022.1001334 36185697 PMC9521368

[66] Jing N Wang L Zhuang H Jiang G Liu Z. Ultrafine Jujube Powder Enhances the Infiltration of Immune Cells during Anti-PD-L1 Treatment against Murine Colon Adenocarcinoma. Cancers (Basel). (2021) 13. doi: 10.3390/cancers13163987 PMC8394940 34439144

[67] Taechakulwanijya N Weerapreeyakul N Barusrux S Siriamornpun S. Apoptosis-inducing effects of jujube (Zăo) seed extracts on human Jurkat leukemia T cells. Chin Med. (2016) 11:15. doi: 10.1186/s13020-016-0085-x 27042202 PMC4818408

[68] Lee D Kang KB Kim HW Park JS Hwang GS Kang KS. Unique triterpenoid of jujube root protects cisplatin-induced damage in kidney epithelial LLC-PK1 cells *via* autophagy regulation. Nutrients. (2020) 12. doi: 10.3390/nu12030677 PMC7146250 32131519

[69] Shin M Lee B-M Kim O Tran HNK Lee S Hwangbo C. Triterpenoids from Ziziphus jujuba induce apoptotic cell death in human cancer cells through mitochondrial reactive oxygen species production. Food Funct. (2018) 9:3895–905. doi: 10.1039/c8fo00526e 29968885

[70] Mitsuhashi Y Furusawa Y Aradate T Zhao Q-L Moniruzzaman R Kanamori M. 3-O-trans-p-coumaroyl-alphitolic acid, a triterpenoid from Zizyphus jujuba, leads to apoptotic cell death in human leukemia cells through reactive oxygen species production and activation of the unfolded protein response. PloS One. (2017) 12:e0183712. doi: 10.1371/journal.pone.0183712 28832644 PMC5568338

[71] Jia M-M Li Y-Q Xu K-Q Zhang Y-Y Tan S-M Zhang Q. Jujuboside B promotes the death of acute leukemia cell in a RIPK1/RIPK3/MLKL pathway-dependent manner. Eur J Pharmacol. (2020) 876:173041. doi: 10.1016/j.ejphar.2020.173041 32142769

[72] Zhang P Lai X Zhu M-H Shi J Pan H Huang Y. Jujuboside B suppresses angiogenesis and tumor growth *via* blocking VEGFR2 signaling pathway. Heliyon. (2023) 9:e17072. doi: 10.1016/j.heliyon.2023.e17072 37484305 PMC10361242

[73] Yang Z Cai W Chen Y Guo Z Xiao Z Zhou T. Jujuboside B reverse CUMS-promoted tumor progression *via* blocking PI3K/akt and MAPK/ERK and dephosphorylating CREB signaling. J Immunol Res. (2022) 2022:5211368. doi: 10.1155/2022/5211368 36254198 PMC9569198

[74] Guo L Liang Y Wang S Li L Cai L Heng Y. Jujuboside B inhibits the proliferation of breast cancer cell lines by inducing apoptosis and autophagy. Front Pharmacol. (2021) 12:668887. doi: 10.3389/fphar.2021.668887 34630073 PMC8497973

[75] Wang W Huang Q Chen Y Huang Z Huang Y Wang Y. The novel FAT4 activator jujuboside A suppresses NSCLC tumorigenesis by activating HIPPO signaling and inhibiting YAP nuclear translocation. Pharmacol Res. (2021) 170:105723. doi: 10.1016/j.phrs.2021.105723 34116210

[76] Shi Q Li X Zhu D Jiang J Li X. Comprehensive analysis of antibacterial and anti-hepatoma activity of metabolites from jujube fruit. Food Biosci. (2022) 47:101511. doi: 10.1016/j.fbio.2021.101511

[77] Kim Y Oh J Jang CH Lim JS Lee JS Kim J-S. *In vivo* anti-inflammatory potential of viscozyme(®)-treated jujube fruit. Foods. (2020) 9. doi: 10.3390/foods9081033 PMC7466189 32752184

[78] Wang B Hui Y Liu L Zhao A Chiou Y-S Zhang F. Optimized extraction of phenolics from jujube peel and their anti-inflammatory effects in LPS-stimulated murine macrophages. J Agric Food Chem. (2019) 67:1666–73. doi: 10.1021/acs.jafc.8b06309 30629413

[79] Cheng N Du B Wang Y Gao H Cao W Zheng J. Antioxidant properties of jujube honey and its protective effects against chronic alcohol-induced liver damage in mice. Food Funct. (2014) 5:900–8. doi: 10.1039/c3fo60623f 24603671

[80] Cardano M Tribioli C Prosperi E. Targeting proliferating cell nuclear antigen (PCNA) as an effective strategy to inhibit tumor cell proliferation. Curr Cancer Drug Targets. (2020) 20:240–52. doi: 10.2174/1568009620666200115162814 31951183

[81] Zare H Nabavizdeh SH Jaladat AM Zarshenas MM Moghtaderi M Basirat A. The added-on of ziziphus jujube syrup in the treatment of chronic spontaneous urticaria resistant to standard-dose of secondary-generation H(1) antihistamine: A double-blind randomized clinical trial. Iran J Med Sci. (2023) 48:582–90. doi: 10.30476/IJMS.2023.95531.2690 PMC10715122 38094286

[82] Xu X Lai Y Hua Z-C. Apoptosis and apoptotic body: disease message and therapeutic target potentials. Biosci Rep. (2019) 39:BSR20180992. doi: 10.1042/BSR20180992 30530866 PMC6340950

[83] Ataş MN Ertuğrul B İplik ES Çakmakoğlu B Ergen A. Effects of betulinic acid on AKT/mTOR pathway in renal cell carcinoma. Turk J Urol. (2022) 48:58–63. doi: 10.5152/tud.2022.21276 35118990 PMC9612733

[84] Zhang Y He N Zhou X Wang F Cai H Huang SH. Betulinic acid induces autophagy-dependent apoptosis *via* Bmi-1/ROS/AMPK-mTOR-ULK1 axis in human bladder cancer cells. Aging (Albany NY). (2021) 13:21251–67. doi: 10.18632/aging.203441 PMC8457576 34510030

[85] Ou H-L Hoffmann R González-López C Doherty GJ Korkola JE Muñoz-Espín D. Cellular senescence in cancer: from mechanisms to detection. Mol Oncol. (2021) 15:2634–71. doi: 10.1002/1878-0261.12807 PMC8486596 32981205

[86] Boutelle AM Attardi LD. p53 and tumor suppression: it takes a network. Trends Cell Biol. (2021) 31:298–310. doi: 10.1016/j.tcb.2020.12.011 33518400 PMC7954925

[87] Kang DY Sp N Jang K-J Jo ES Bae SW Yang YM. Antitumor effects of natural bioactive ursolic acid in embryonic cancer stem cells. J Oncol. (2022) 2022:6737248. doi: 10.1155/2022/6737248 35222644 PMC8866021

[88] Hopkins JL Lan L Zou L. DNA repair defects in cancer and therapeutic opportunities. Genes Dev. (2022) 36:278–93. doi: 10.1101/gad.349431.122 PMC8973847 35318271

[89] Wang S Chang X Zhang J Li J Wang N Yang B. Ursolic acid inhibits breast cancer metastasis by suppressing glycolytic metabolism *via* activating SP1/caveolin-1 signaling. Front Oncol. (2021) 11:745584. doi: 10.3389/fonc.2021.745584 34568078 PMC8457520

[90] Debnath J Gammoh N Ryan KM. Autophagy and autophagy-related pathways in cancer. Nat Rev Mol Cell Biol. (2023) 24:560–75. doi: 10.1038/s41580-023-00585-z PMC9980873 36864290

[91] Fogde DL Xavier CPR Balnytė K Holland LKK Stahl-Meyer K Dinant C. Ursolic acid impairs cellular lipid homeostasis and lysosomal membrane integrity in breast carcinoma cells. Cells. (2022) 11. doi: 10.3390/cells11244079 PMC9776894 36552844

[92] Potočnjak I Šimić L Vukelić I Batičić L Domitrović R. Oleanolic acid induces HCT116 colon cancer cell death through the p38/FOXO3a/Sirt6 pathway. Chem Biol Interact. (2022) 363:110010. doi: 10.1016/j.cbi.2022.110010 35690101

[93] Ruan J Han Y Kennedy JF Jiang H Cao H Zhang Y. A review on polysaccharides from jujube and their pharmacological activities. Carbohydr Polymer Technol Appl. (2022) 3:100220. doi: 10.1016/j.carpta.2022.100220

[94] Dash SK Chattopadhyay S Tripathy S Dash SS Das B Mandal D. Self-assembled betulinic acid augments immunomodulatory activity associates with IgG response. Biomed Pharmacother. (2015) 75:205–17. doi: 10.1016/j.biopha.2015.07.033 26256937

[95] Liu G Liu X Zhang Y Zhang F Wei T Yang M. Hepatoprotective effects of polysaccharides extracted from Zizyphus jujube cv. Huanghetanzao. Int J Biol Macromol. (2015) 76:169–75. doi: 10.1016/j.ijbiomac.2015.01.061 25709018

[96] Dong X-N Li M-T Gu H-Y Zhu Y Gu X. Advances in pharmacological effects of jujuboside B. Zhongguo Zhong Yao Za Zhi. (2023) 48:4295–301. doi: 10.19540/j.cnki.cjcmm.20230320.701 37802856

[97] Mohebbati R Kamkar-Del Y Shafei MN. Effect of aqueous and ethyl acetate fractions of ziziphus jujuba mill extract on cardiovascular responses in hypertensive rats. Malays J Med Sci. (2020) 27:43–52. doi: 10.21315/mjms2020.27.3.5 PMC7337946 32684805

[98] Chen L Zhang X Hu C Zhang Y Zhang L Kan J. Regulation of GABA(A) and 5-HT receptors involved in anxiolytic mechanisms of jujube seed: A system biology study assisted by UPLC-Q-TOF/MS and RT-qPCR method. Front Pharmacol. (2020) 11:1320. doi: 10.3389/fphar.2020.01320 PMC7593408 33178009

[99] Liu J Zhai W-M Yang Y-X Shi J-L Liu Q-T Liu G-L. GABA and 5-HT systems are implicated in the anxiolytic-like effect of spinosin in mice. Pharmacol Biochem Behav. (2015) 128:41–9. doi: 10.1016/j.pbb.2014.11.003 25449359

[100] Yang M Wang H Zhang YL Zhang F Li X Kim S-D. The herbal medicine suanzaoren (Ziziphi spinosae semen) for sleep quality improvements: A systematic review and meta-analysis. Integr Cancer Ther. (2023) 22:15347354231162080. doi: 10.1177/15347354231162080 37014010 PMC10084578

[101] Bian Z-H Zhang W-M Tang J-Y Fei Q-Q Hu M-M Chen X-W. Effective substance and mechanism of Ziziphi Spinosae Semen extract in treatment of insomnia based on serum metabolomics and network pharmacology. Zhongguo Zhong Yao Za Zhi. (2022) 47:188–202. doi: 10.19540/j.cnki.cjcmm.20210922.702 35178926

[102] Qiao T Wang Y Liang K Zheng B Ma J Li F. Effects of the Radix Ginseng and Semen Ziziphi Spinosae drug pair on the GLU/GABA-GLN metabolic cycle and the intestinal microflora of insomniac rats based on the brain-gut axis. Front Pharmacol. (2022) 13:1094507. doi: 10.3389/fphar.2022.1094507 36618926 PMC9811267

[103] Xu D Xiao J Jiang D Liu Y Gou Z Li J. Inhibitory effects of a water-soluble jujube polysaccharide against biofilm-forming oral pathogenic bacteria. Int J Biol Macromol. (2022) 208:1046–62. doi: 10.1016/j.ijbiomac.2022.03.196 35378158

[104] Miao W Sheng L Yang T Wu G Zhang M Sun J. The impact of flavonoids-rich Ziziphus jujuba Mill. Extract on Staphylococcus aureus biofilm formation. BMC Complement Med Ther. (2020) 20:187. doi: 10.1186/s12906-020-2833-9 32552790 PMC7301566

[105] Das U Manna K Adhikary A Mishra S Saha KD Sharma RD. Ferulic acid enhances the radiation sensitivity of lung and liver carcinoma cells by collapsing redox homeostasis: mechanistic involvement of Akt/p38 MAPK signalling pathway. Free Radic Res. (2019) 53:944–67. doi: 10.1080/10715762.2019.1655559 31576765

[106] Ji X Hou C Gao Y Xue Y Yan Y Guo X. Metagenomic analysis of gut microbiota modulatory effects of jujube (Ziziphus jujuba Mill.) polysaccharides in a colorectal cancer mouse model. Food Funct. (2020) 11:163–73. doi: 10.1039/c9fo02171j 31830158

[107] Xu Y Wang B Zhang M Zhang J Li Y Jia P. Carbon dots as a potential therapeutic agent for the treatment of cancer-related anemia. Adv Mater. (2022) 34:e2200905. doi: 10.1002/adma.202200905 35294781

[108] Dash SK Chattopadhyay S Ghosh T Dash SS Tripathy S Das B. Self-assembled betulinic acid protects doxorubicin induced apoptosis followed by reduction of ROS-TNF-α-caspase-3 activity. BioMed Pharmacother. (2015) 72:144–57. doi: 10.1016/j.biopha.2015.04.017 26054689

[109] Huang X Kojima-Yuasa A Xu S Norikura T Kennedy DO Hasuma T. Green tea extract enhances the selective cytotoxic activity of Zizyphus jujuba extracts in HepG2 cells. Am J Chin Med. (2008) 36:729–44. doi: 10.1142/S0192415X08006193 18711770

