# Supplementary material for: Review: performance of jujube and its extracts in cancer: therapeutic, toxicity-reducing and potentiating effects
Source: Front Oncol. 2025 Jan 29;15:1489974. doi: 10.3389/fonc.2025.1489974 (PMC11814214; doi:10.3389/fonc.2025.1489974)
Supplement: Supplementary file 1 [file Table1.docx]

| Name | PubChem CID | Molecular Formula | Canonical SMILES | Structure |
| --- | --- | --- | --- | --- |
| stepharine | 98455 | C_18_H_19_NO_3_ | COC1=C(C2=C3C(CC24C=CC(=O)C=C4)NCCC3=C1)OC | 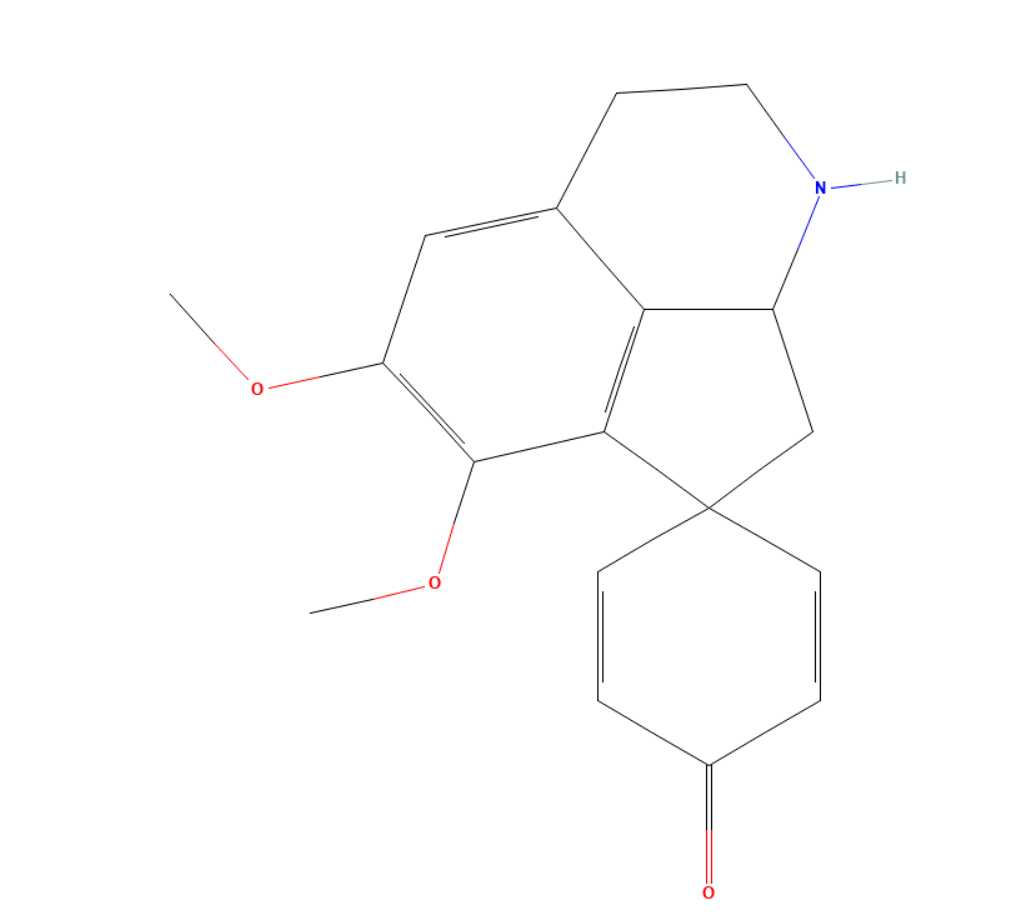 |
| zizyphus saponin I_qt | 102475150 | C_51_H_82_O_22_ | CC1CCC2(C(C3C(O2)CC4C3(CCC5C4CC=C6C5(CCC(C6)OC7C(C(C(C(O7)CO)OC8C(C(C(C(O8)CO)O)OC9C(C(C(C(O9)CO)O)O)O)O)O)OC2C(C(C(C(O2)C)O)O)O)C)C)C)OC1 | 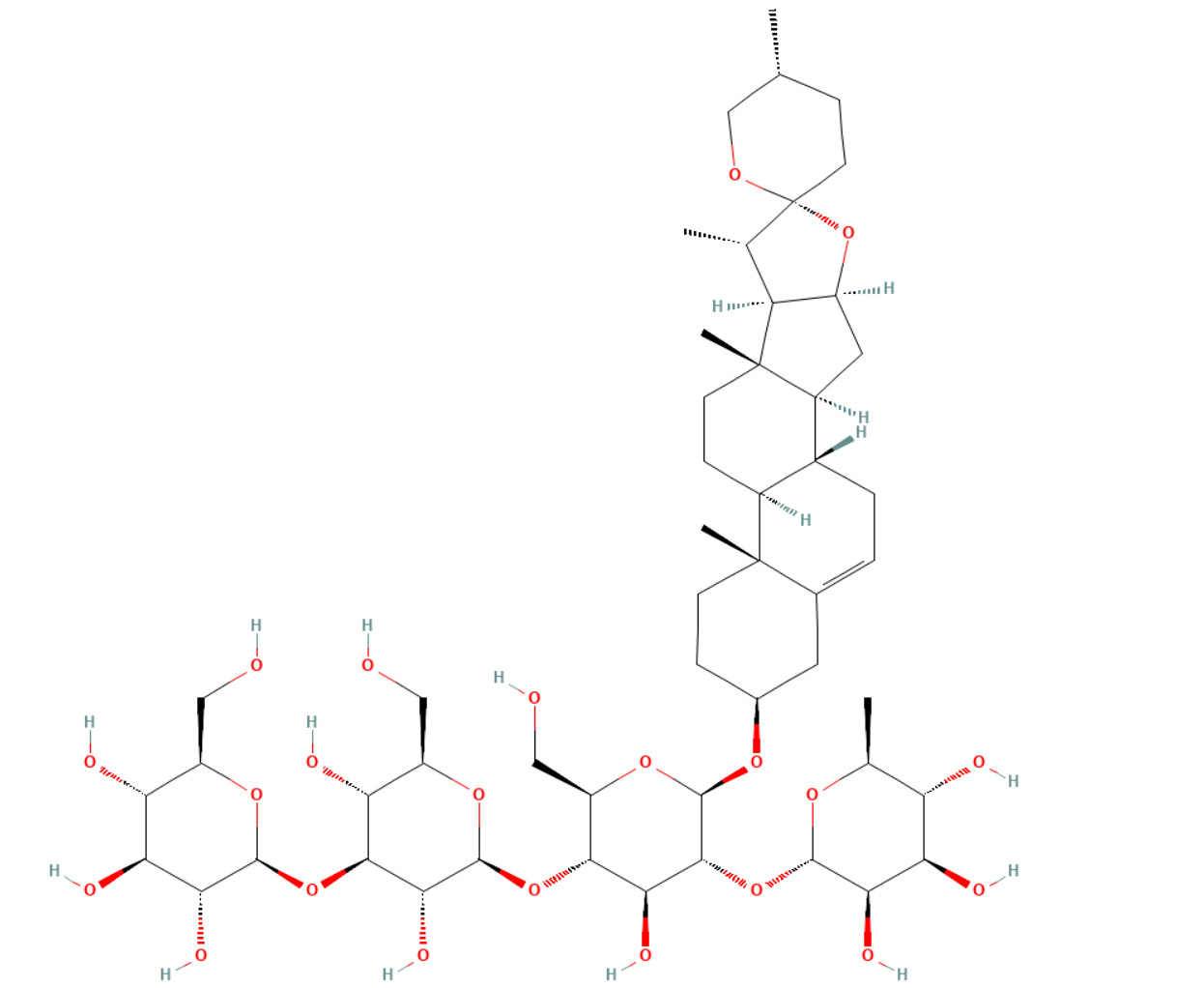 |
| Jujubasaponin V_qt | 274197523 | C_48_H_78_O_18_ | C[C@@H]1O[C@@H](O[C@H]2[C@H](OC3CC[C@@]4(C)[C@@H](CC[C@]5(C)[C@@H]4CC[C@@H]4[C@H]6[C@@](C)(O)[C@H](CC=C(C)C)O[C@]76C[C@@]54CO7)C3(C)C)O[C@H](CO)[C@@H](O)[C@@H]2O[C@@H]2O[C@H](CO)[C@@H](O)[C@H](O)[C@H]2O)[C@H](O)[C@H](O)[C@H]1O | 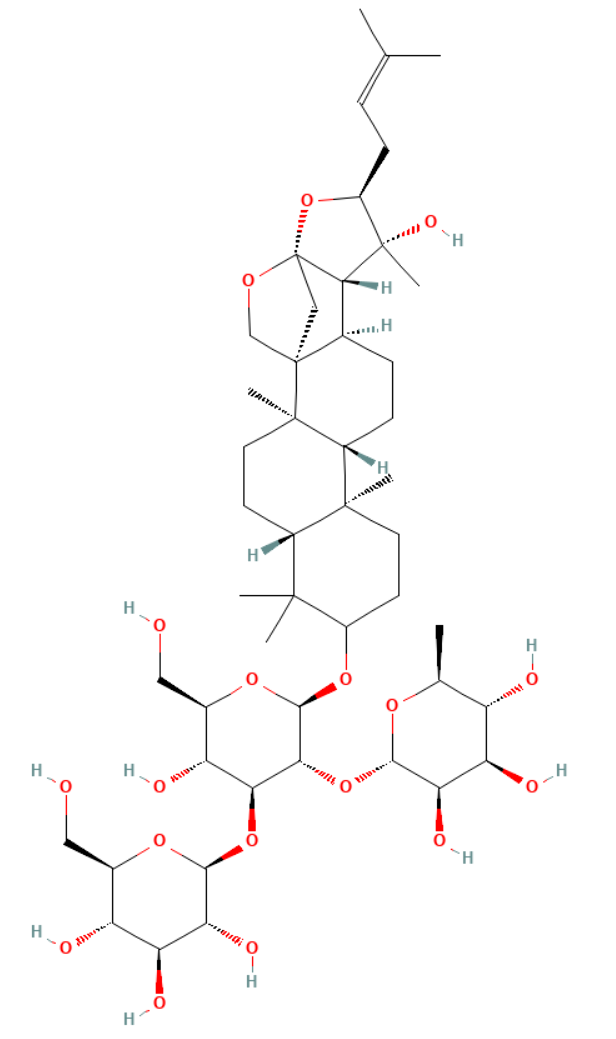 |
| Mauritine D | 6443026 | C_33_H_51_NO_5_ | CCC(C)C1C(=O)NC=CC2=CC=C(C=C2)OC3CCN(C3C(=O)N1)C(=O)C(CC(C)C)NC(=O)C(C(C)CC)N(C)C | 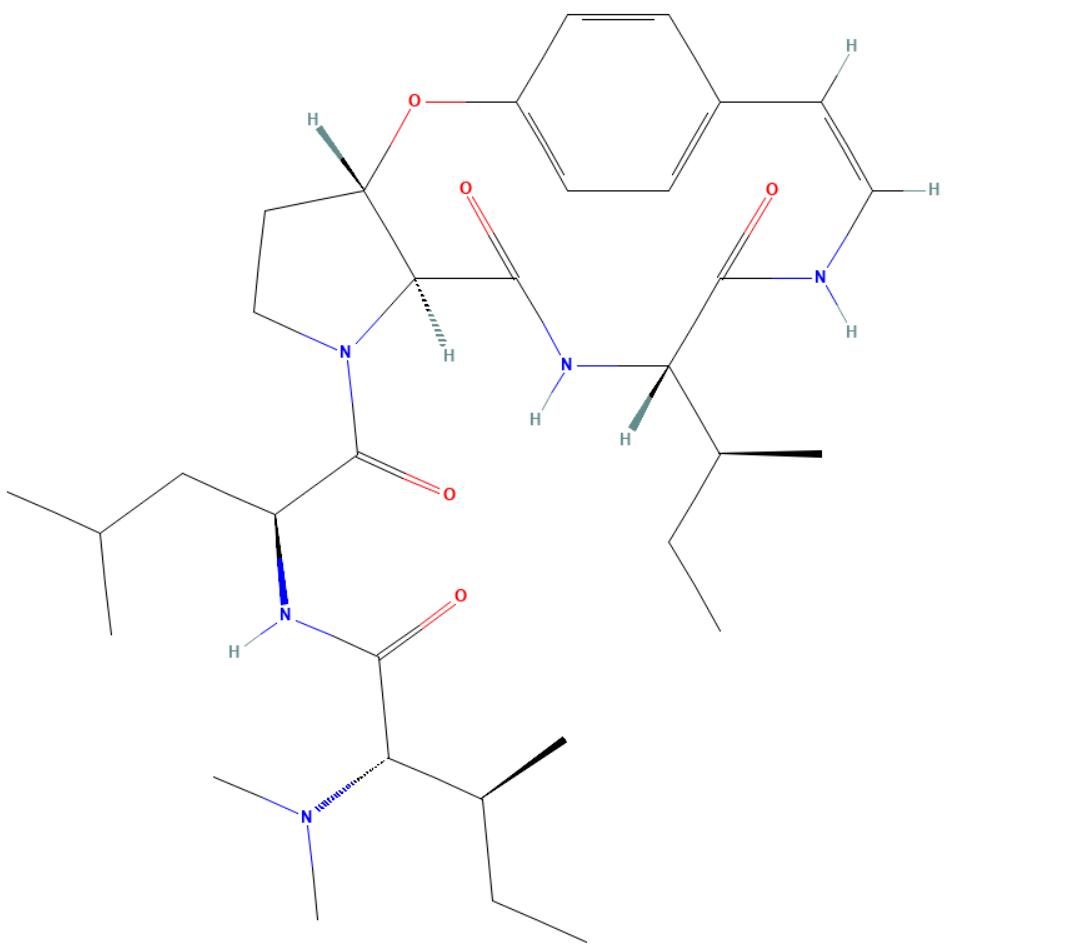 |
| berberine | 2353 | C_20_H_18_NO_4_ | COC1=C(C2=C[N+]3=C(C=C2C=C1)C4=CC5=C(C=C4CC3)OCO5)OC | 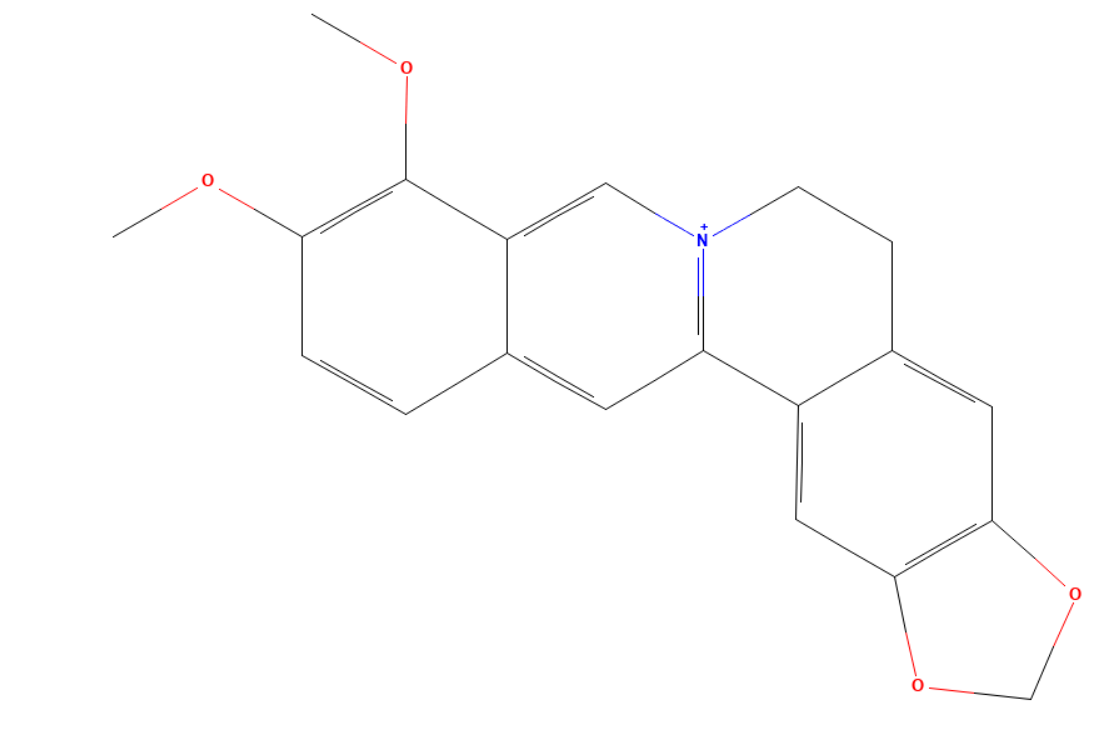 |
| (S)-Coclaurine | 160487 | C_17_H_19_NO_3_ | COC1=C(C=C2C(NCCC2=C1)CC3=CC=C(C=C3)O)O | 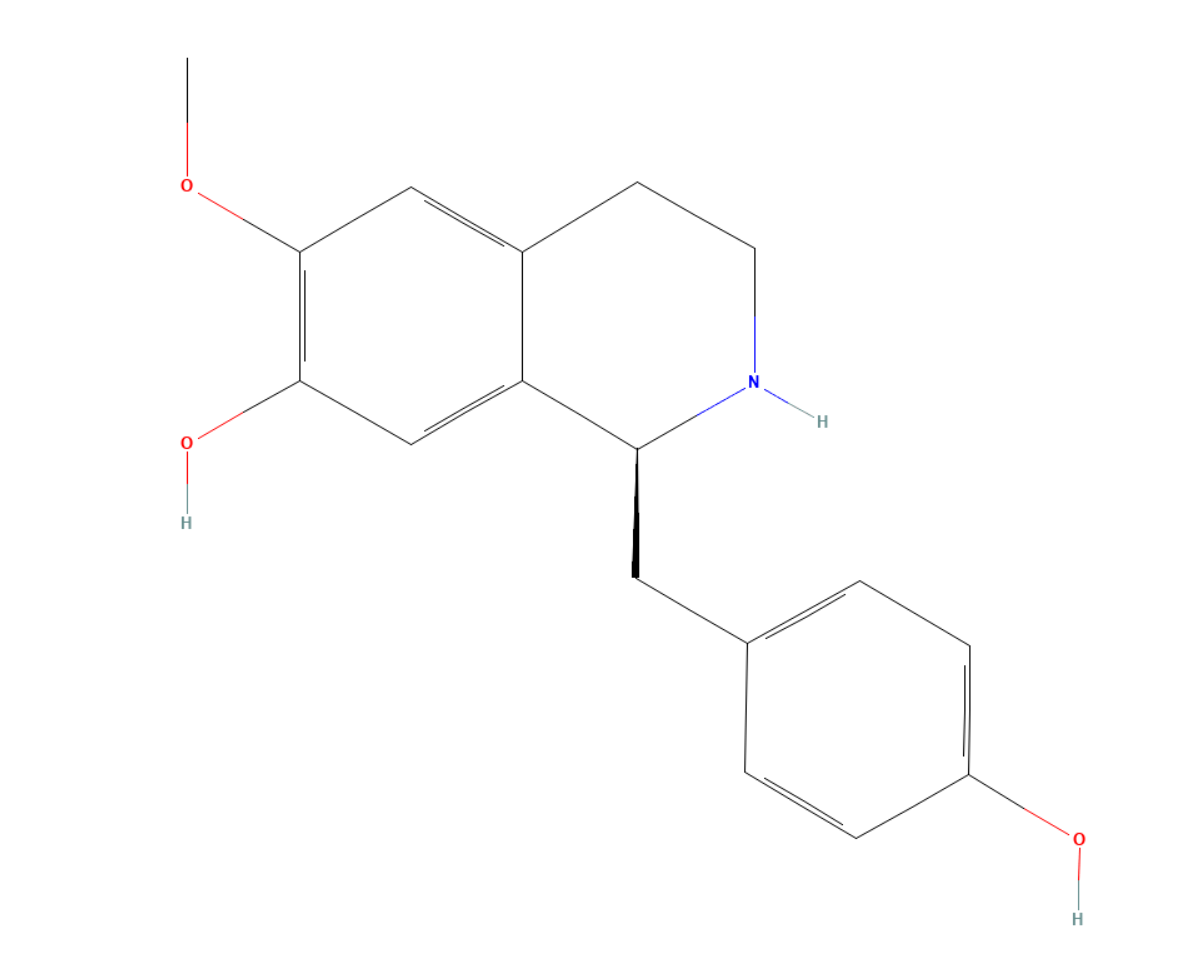 |
| Mairin | 64971 | C_30_H_48_O_3_ | CC(=C)C1CCC2(C1C3CCC4C5(CCC(C(C5CCC4(C3(CC2)C)C)(C)C)O)C)C(=O)O | 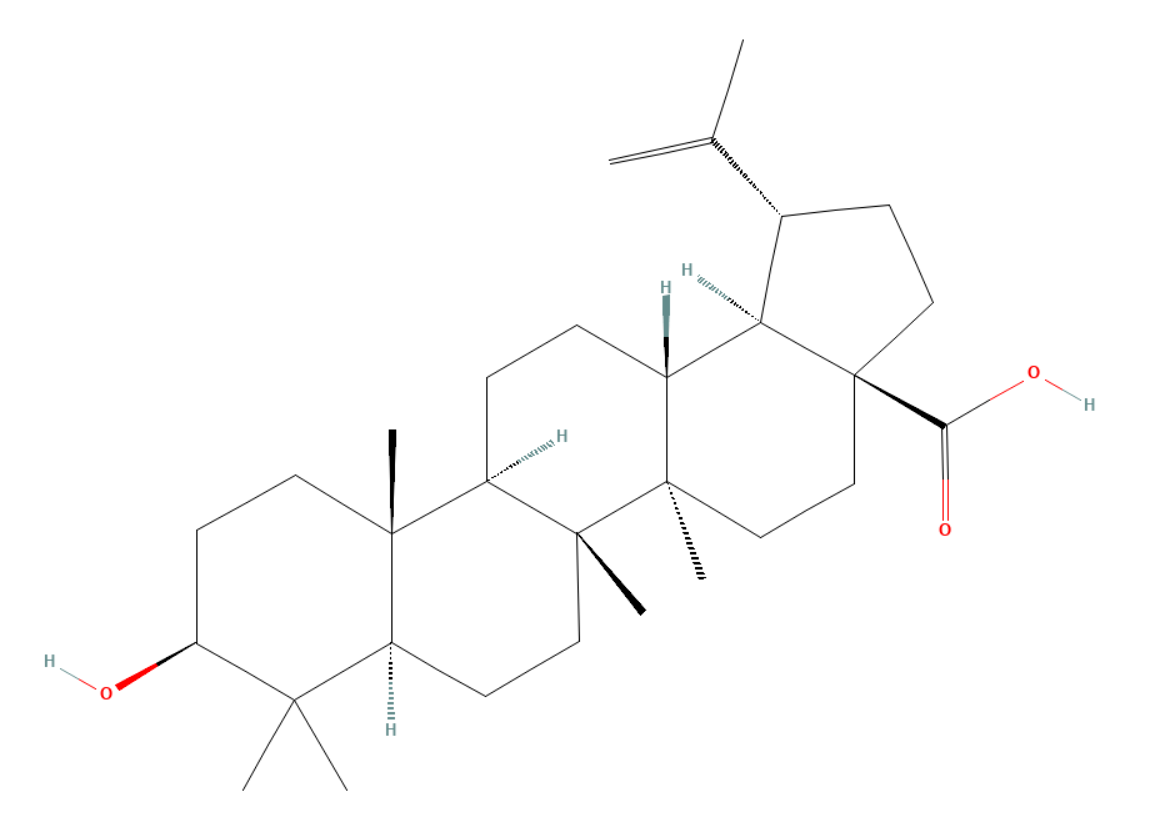 |
| Stigmasterol | 5280794 | C_29_H_48_O | CCC(C=CC(C)C1CCC2C1(CCC3C2CC=C4C3(CCC(C4)O)C)C)C(C)C | 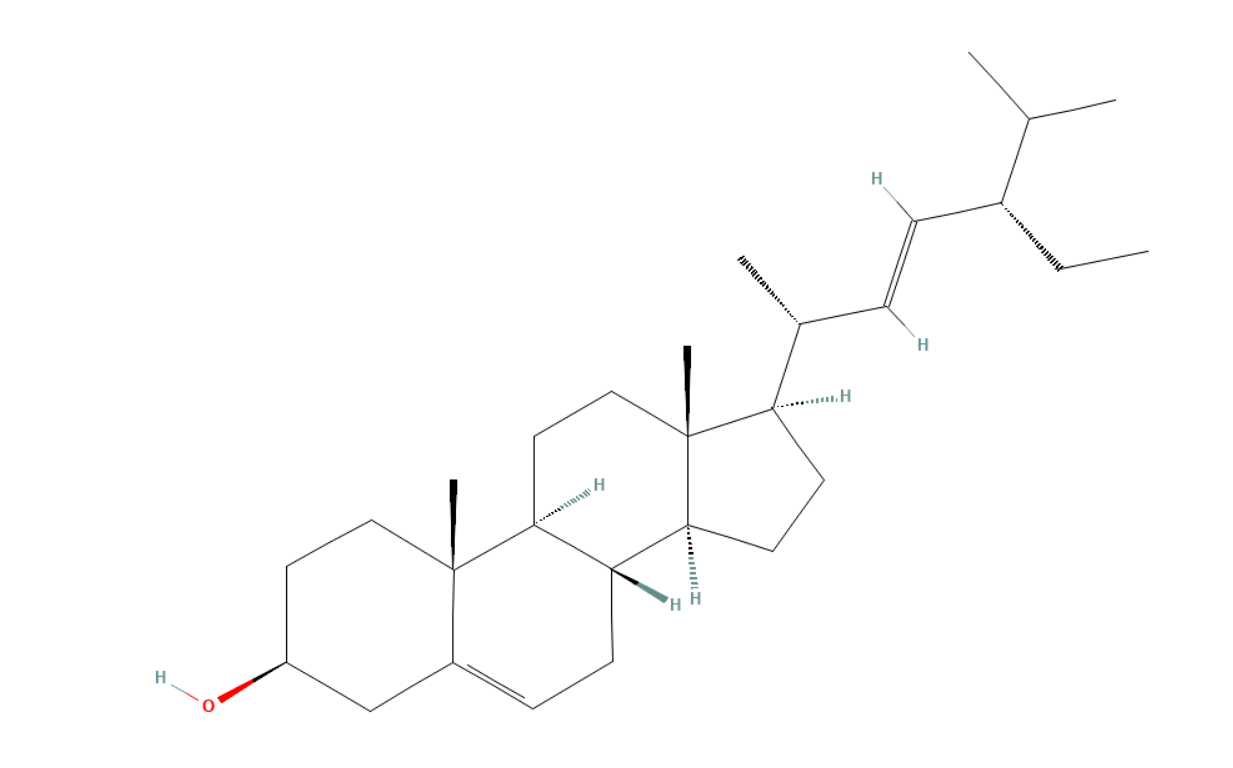 |
| beta-sitosterol | 222284 | C_29_H_50_O | CCC(CCC(C)C1CCC2C1(CCC3C2CC=C4C3(CCC(C4)O)C)C)C(C)C | 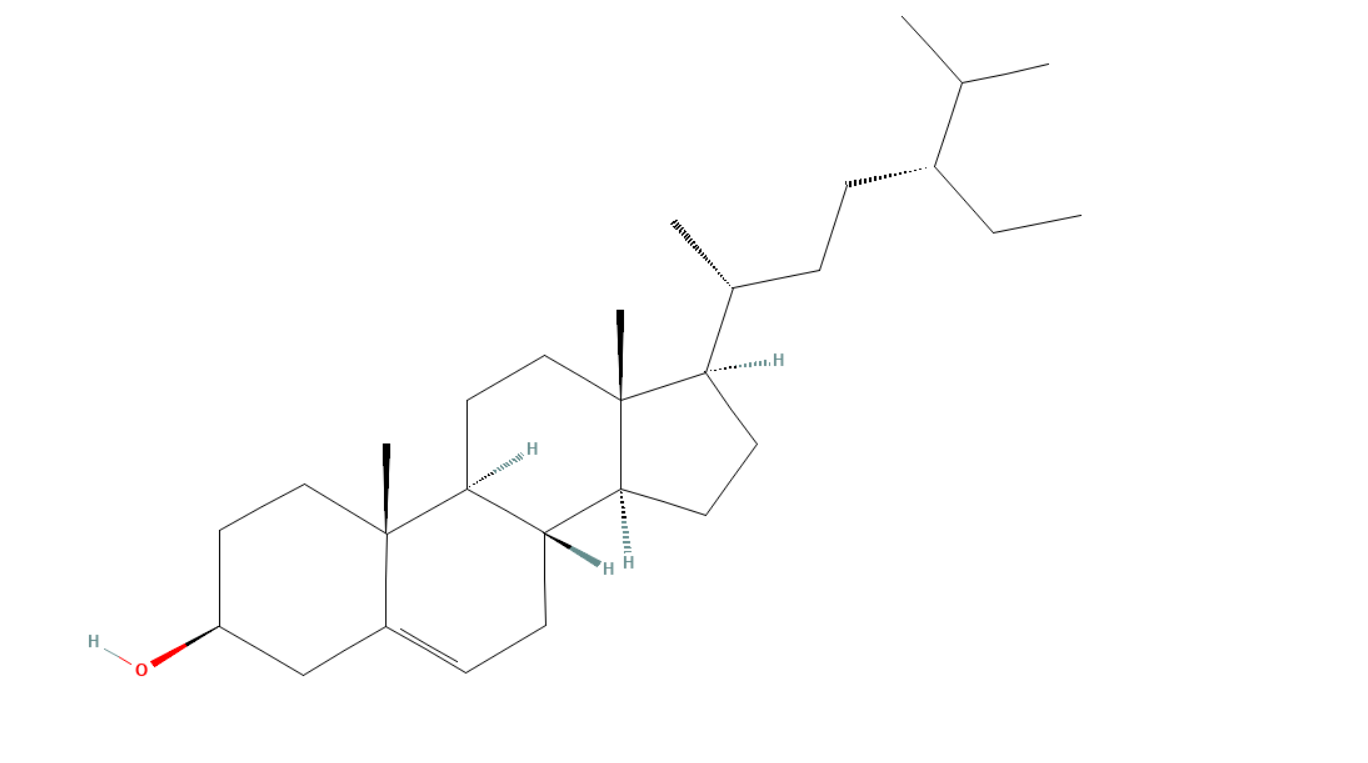 |
| Ruvoside_qt | 101650325 | C_30_H_46_O_9_ | CC1C(C(C(C(O1)OC2CCC3(C(C2)CCC4C3CCC5(C4(CCC5C6=CC(=O)OC6)O)C)CO)O)OC)O | 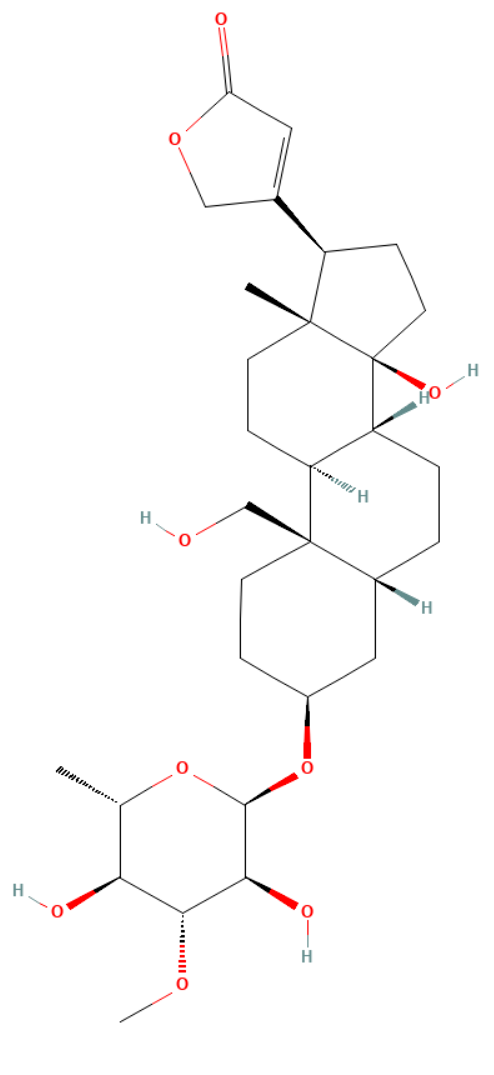 |
| (+)-catechin | 9064 | C_15_H_14_O_6_ | C1C(C(OC2=CC(=CC(=C21)O)O)C3=CC(=C(C=C3)O)O)O | 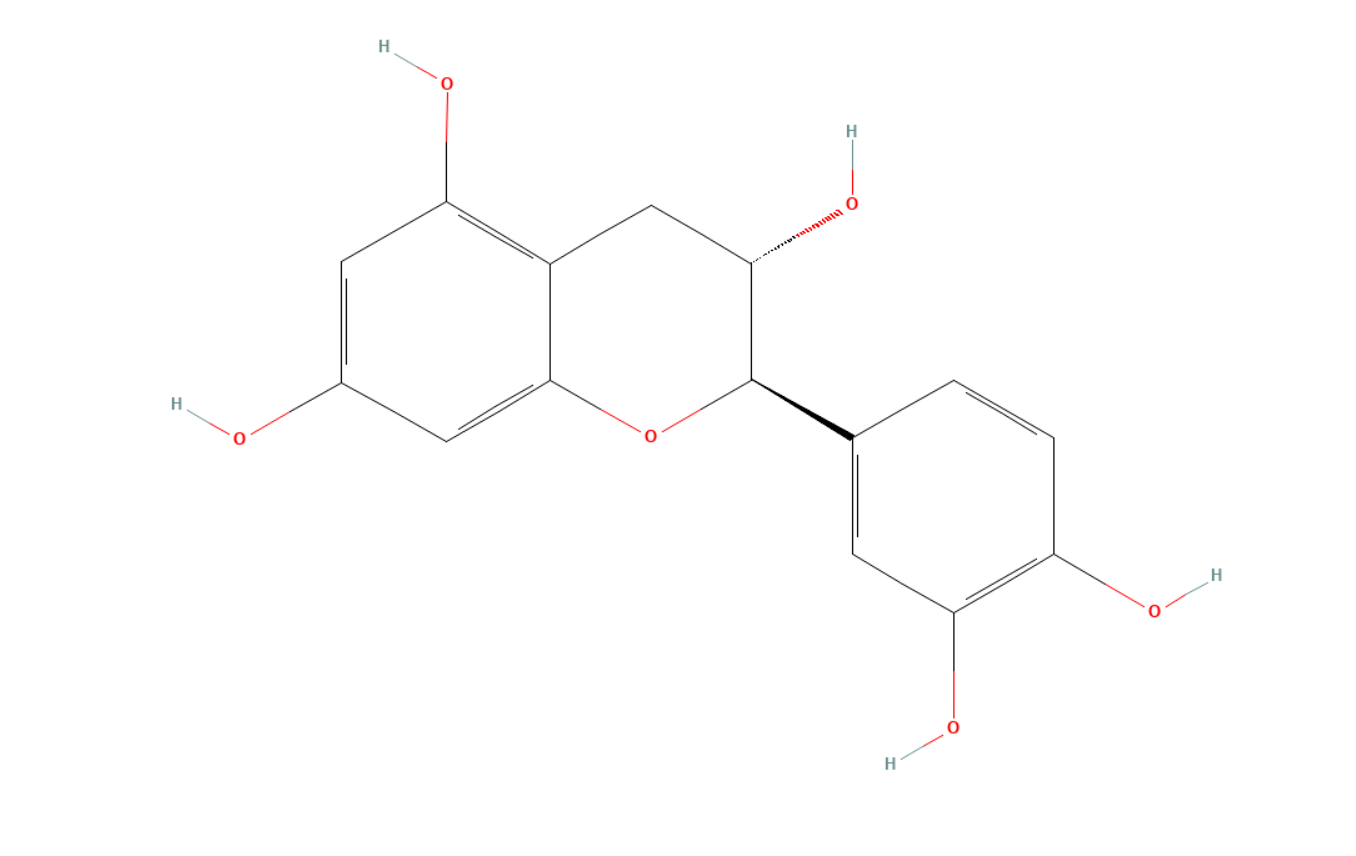 |
| Stepholidine | 6917970 | C_19_H_21_NO_4_ | COC1=C(C=C2C3CC4=C(CN3CCC2=C1)C(=C(C=C4)O)OC)O | 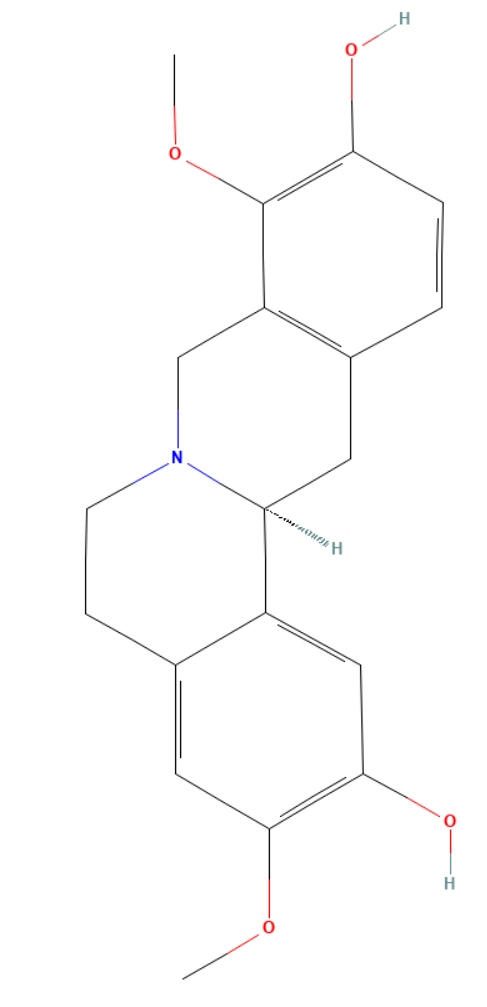 |
| Nuciferin | 10146 | C_19_H_21_NO_2_ | CN1CCC2=CC(=C(C3=C2C1CC4=CC=CC=C43)OC)OC | 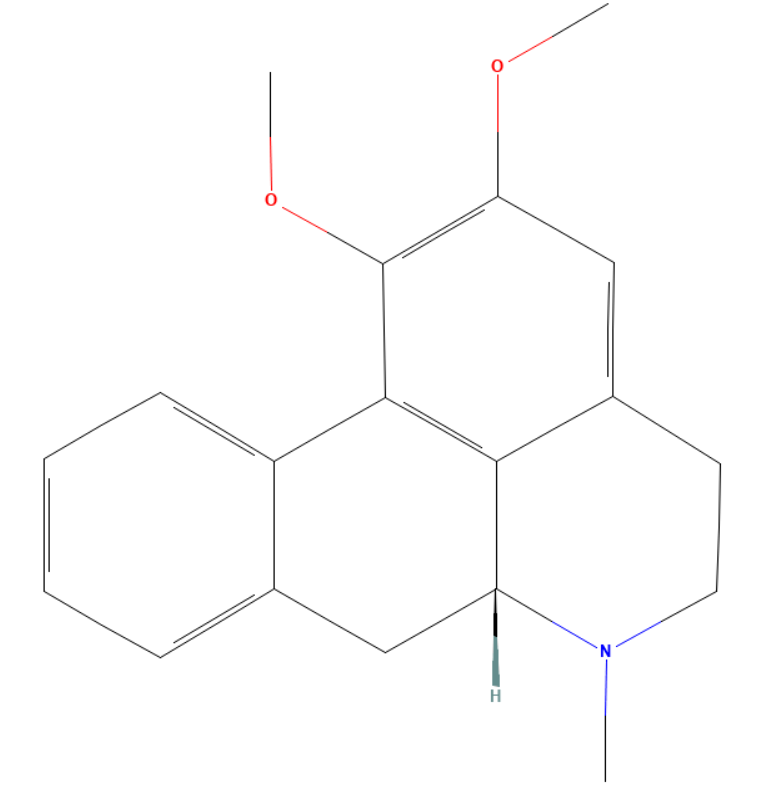 |
| Fumarine | 4970 | C_20_H_19_NO_5_ | CN1CCC2=CC3=C(C=C2C(=O)CC4=C(C1)C5=C(C=C4)OCO5)OCO3 | 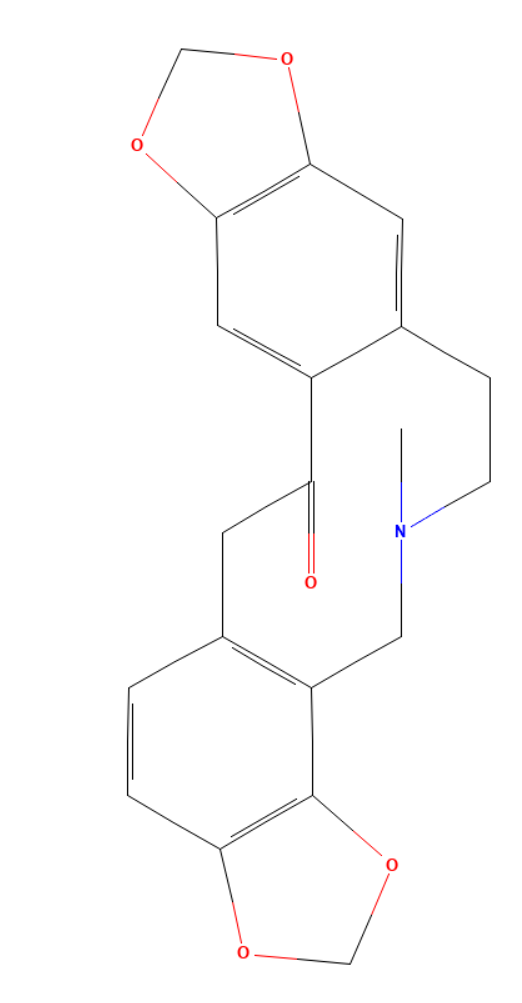 |
| beta-carotene | 5280489 | C_40_H_56_ | CC1=C(C(CCC1)(C)C)C=CC(=CC=CC(=CC=CC=C(C)C=CC=C(C)C=CC2=C(CCCC2(C)C)C)C)C | 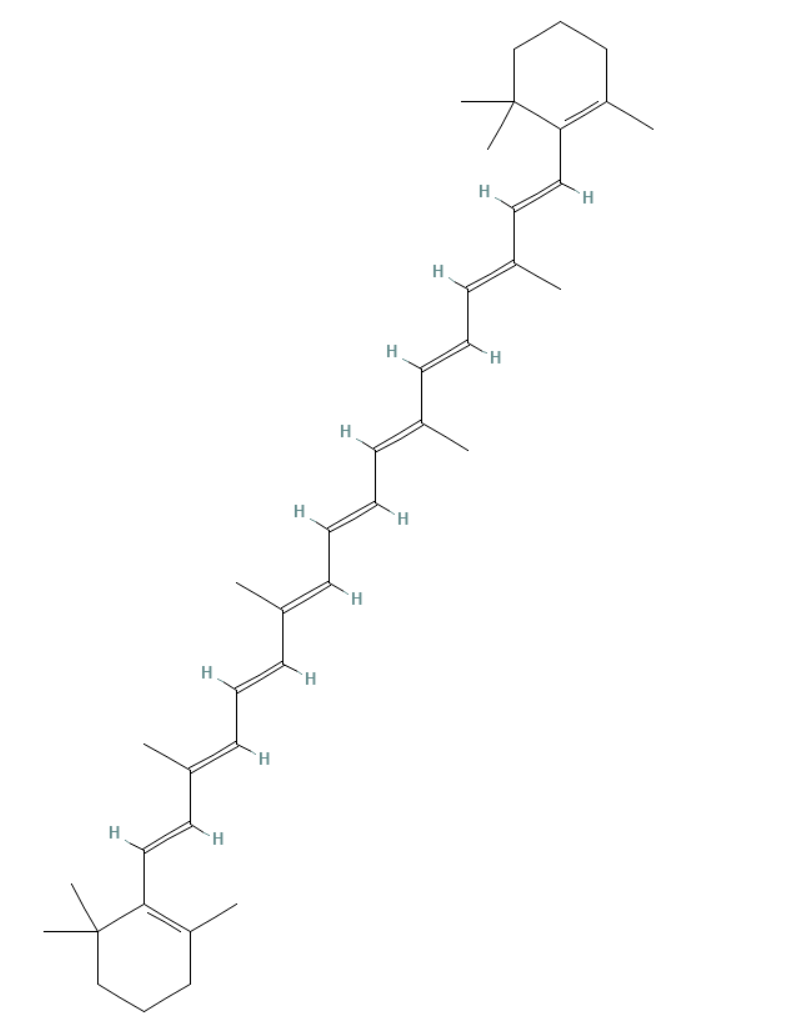 |
| (-)-catechin | 73160 | C_15_H_14_O_6_ | C1C(C(OC2=CC(=CC(=C21)O)O)C3=CC(=C(C=C3)O)O)O | 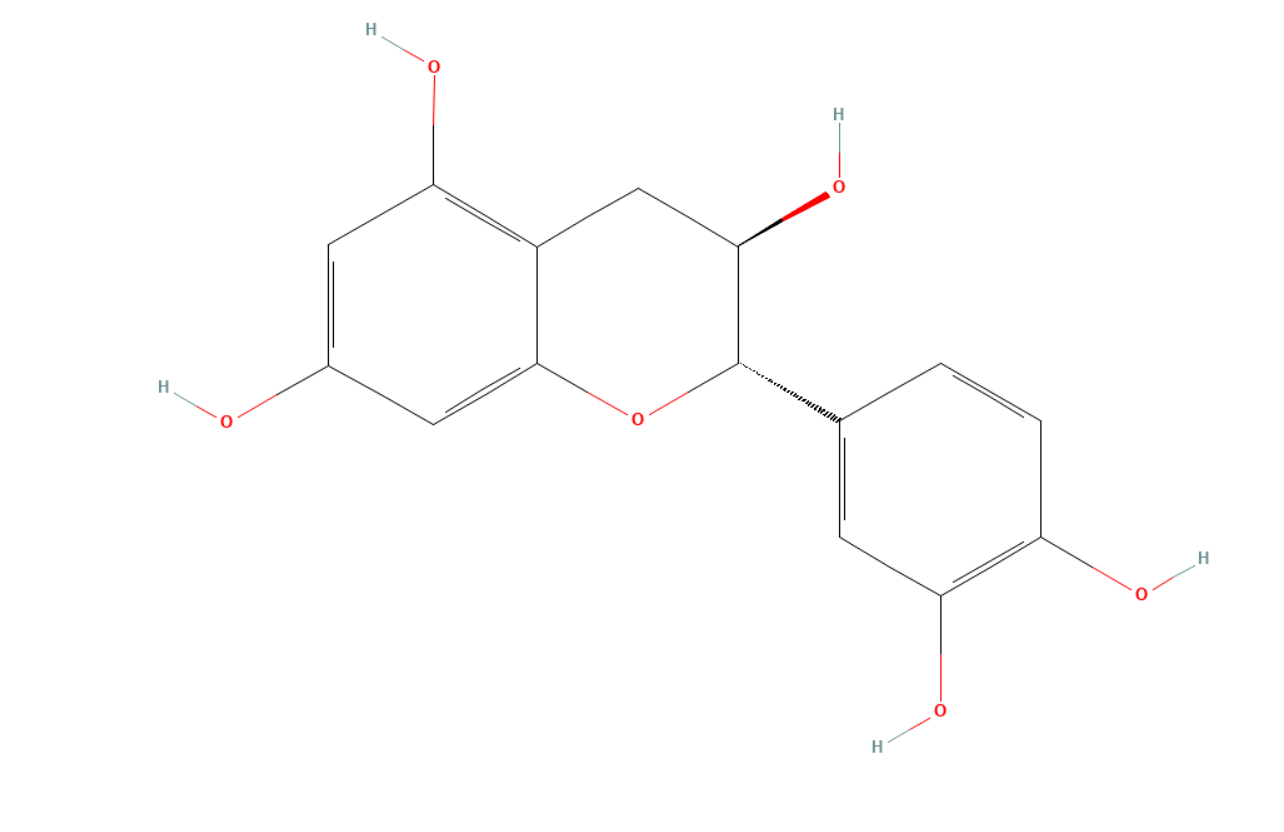 |
| quercetin | 5280343 | C_15_H_10_O_7_ | C1=CC(=C(C=C1C2=C(C(=O)C3=C(C=C(C=C3O2)O)O)O)O)O | 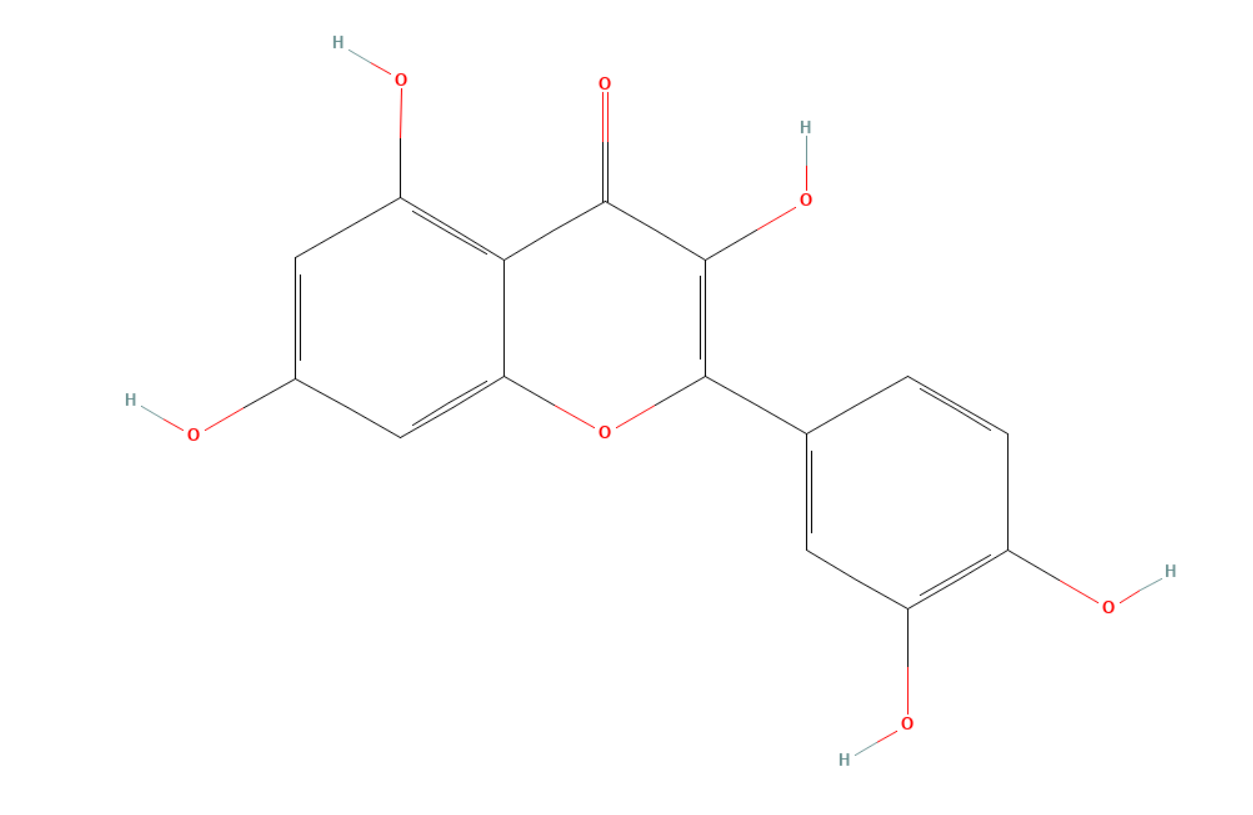 |
